# Adaptive evolution driving the young duplications in six Rosaceae species

**DOI:** 10.1186/s12864-021-07422-7

**Published:** 2021-02-09

**Authors:** Yan Zhong, Xiaohui Zhang, Qinglong Shi, Zong-Ming Cheng

**Affiliations:** 1 College of Horticulture, Nanjing Agricultural University, Nanjing, 210095 China; 2 School of Life Science, Nanjing University, Nanjing, 210023 China

**Keywords:** Young duplication, Rosaceae species, Species-specific expansion, Lineage-specific expansion, Environmental stresses, Adaptive evolution

## Abstract

**Background:**

In plant genomes, high proportions of duplicate copies reveals that gene duplications play an important role in the evolutionary processes of plant species. A series of gene families under positive selection after recent duplication events in plant genomes indicated the evolution of duplicates driven by adaptive evolution. However, the genome-wide evolutionary features of young duplicate genes among closely related species are rarely reported.

**Results:**

In this study, we conducted a systematic survey of young duplicate genes at genome-wide levels among six Rosaceae species, whose whole-genome sequencing data were successively released in recent years. A total of 35,936 gene families were detected among the six species, in which 60.25% were generated by young duplications. The 21,650 young duplicate gene families could be divided into two expansion types based on their duplication patterns, species-specific and lineage-specific expansions. Our results showed the species-specific expansions advantaging over the lineage-specific expansions. In the two types of expansions, high-frequency duplicate domains exhibited functional preference in response to environmental stresses.

**Conclusions:**

The functional preference of the young duplicate genes in both the expansion types showed that they were inclined to respond to abiotic or biotic stimuli. Moreover, young duplicate genes under positive selection in both species-specific and lineage-specific expansions suggested that they were generated to adapt to the environmental factors in Rosaceae species.

**Supplementary Information:**

The online version contains supplementary material available at 10.1186/s12864-021-07422-7.

## Background

Gene duplications contribute to the generation of new genetic materials and novel gene functions, which drive the evolution and divergence of genomes and genetic systems [1, 2]. In plant genomes, the frequent occurrence of whole-genome duplications, segmental duplications, and polyploidizations results in masses of duplication loci [3, 4]. The whole-genome duplication (WGD), a sort of gene duplications sharply accelerates the scale of chromosome or the whole genome, but followed by a series of gene loss, gene conversion and so on [5, 6]. For tandem duplication, it might be caused by unequal crossing over leading to the progeny duplicates located adjacently to each other in a cluster intra-chromosome [6, 7]. The tandemly duplicate copies exhibit a coordinated expression mode and increase the divergence distance among themselves [7]. The transposon-related duplication or tansponson-mediated duplication is replicative transposition involved with transposable elements [6]. For example, in *Oryza sativa* (rice) and *Arabidopsis*, approximately 15–62% and 90%, respectively, of the gene loci are estimated to arise from gene duplication [8–10].

The large-scale existence of duplicate genes implies the retention and evolution of duplicates among plant genomes [5]. However, duplicate genes face three long-term fates: nonfunctionalization (or pseudogenization), characterized by one of the copies losing its function; neofunctionalization reflected by one of the copies gaining a novel function; and subfunctionalization exhibited by duplicate copies inheriting parts of the original gene function [5]. Nonfunctionalization/pseudogenization is the most widespread fate of the duplicate copies. However, neofunctionalization is the preservation mechanism to retain them, which is reflected by the positive selection during or after duplicate fixation [1, 11].

The signatures of positive selection acting on duplicate genes, commonly indicating that the duplicates are subject to adaptive evolution, were previously reported in plant genomes. For example, in *Arabidopsis thaliana*, the imprinted gene *MEDEA* (*MEA*) undergoes positive Darwinian selection along with neofunctionalization after duplication [12]; similarly, in *Arabidopsis* and a few grass species, the centromere protein C (*CENP-C*) genes with complex duplicate regions are under positive selective pressure [13]; the chalcone synthase (*CHS*) genes undergo positive selection in *Dendranthema* genomes [14]. Furthermore, a group of young duplicate genes that underwent adaptive evolution were detected in plants, such as the extremely expanded nucleotide-binding site leucine-rich repeat (*NBS-LRR*) genes of *Vitis vinifera*, *Populus trichocarpa*, and the Rosaceae species [15–17]. However, the evolutionary characteristics of young duplicate genes have been rarely reported at genome-wide levels among closely related plant species.

The whole-genome sequencing of *Fragaria vesca*, *Malus x domestica*, *Pyrus communis*, *Prunus persica*, *Rosa chinensis*, and *Rubus occidentalis* provides us an opportunity to investigate the evolution of the recent duplicate genes among the six Rosaceae genomes. The Rosaceae is a large family possessing high economical values, composed by four subfamilies, *Spiraeoideae*, *Rosoideae*, *Maloideae*, and *Prunoideae*. The six species involved in three subfamilies of Rosaceae, covering different evolutionary distances, containing *Rosoideae* (*F. vesca*, *R. chinensis* and *R. occidentalis*), *Maloideae* (*M. x domestica* and *P. communis*) and *Prunoideae* (*P. persica*). The origination of Rosaceae family is predicted during the Late Cretaceous [18]. Nine ancestral chromosomes existing in the ancestral Rosaceae genome, modern Rosaceae genomes are generated after a series of chromosome fission, fusion, and duplications during the evolutionary processes of Rosaceae family [19]. Especially, the genomes of *M. x domestica* and *P. communis* underwent a common recent WGD, but no similar large-scale duplication was reported in the other four genomes [20–26]. In our study, a genome-wide identification and genetic evolution analysis of young duplications were performed among the six diploid genomes. Our results demonstrated that the young duplicates underwent adaptive evolution for acclimatization in the six species.

## Results

### Young duplicate genes in the six Rosaceae species

A total of 35,936 gene families were explored across the six Rosaceae species containing 21,650 young duplicate gene families, which indicated that young duplications occurred in 60.25% of the total gene families (Table 1 and Additional file 1: Table S1). Species-specific and lineage-specific expansions were detected in these young duplicate gene families based on their duplication patterns. The total family number of species-specific expansions (14,988) outdistanced that of lineage-specific expansions (6662). In species-specific expansions, distinct family numbers were found among the six species, such as the most gene families in *M. x domestica* (6184), moderate number in *P. communis* (3122), and the least gene families (791) in *R. occidentalis*. Interestingly, in lineage-specific expansions, there was an extremely high value (6105) in the lineages of *M. x domestica* and *P. communis*, probably because of the close phylogenetic relationship between the two species and the common recent WGD shaping and increasing their genomes [21, 22]. Except in the lineages of *M. x domestica* and *P. communis*, a broad range of family numbers (1 to 149) were detected in lineage-specific expansions. The second largest gene number (149) was observed in the lineages of *F. vesca* and *R. chinensis,* which may be attributed to their close evolutionary relationship. The similar phenomenons were also found in the lineages of *M. x domestica*, *P. communis* and *P. persica* or *R. chinensis* and *R. occidentalis* (Table 1 and Additional file 1: Table S1).

**Table 1:** Number of young duplicate gene families for two types of expansions

Species	Species-specific expansions	Lineage-specific expansions																	
		2 ^a^	2	2	2	2	3	3	3	3	4	4	4	4	5	5	5	5	6
***F. vesca***	1145	+ ^b^	+	+		…^c^	+	+	+	…	+	+	+	…	+	+	+		+
***M. x domestica***	6184	+			+		+				+	+			+	+	+	+	+
***P. communis***	3122		+		+		+	+			+	+	+		+	+		+	+
***P. persica***	1233										+		+		+		+	+	+
***R. chinensis***	2513			+				+	+			+	+		+	+	+	+	+
***R. occidentalis***	791								+							+	+	+	+
		1	4	149	6105	163	16	1	25	150	1	7	1	22	1	3	1	5	7
**Total**	**14,988**	**6662**																	

^a^ These number means the species numbers involved in lineage-specific expansions

^b^ Corresponding species involved in the lineage-specific expansions

^c^ Not all lineage-specific expansions have been shown due to space limitation. The total number of other two-species-lineage-specific expansions is shown in this row (Please see Table S1 for the full version)

For the families belonging to lineage-specific expansions, it is worth mentioning that seven young duplicate gene families included 156 gene members from the lineages of all six species. That is, each of the six species has two or more gene members in each of the seven gene families. To detect the species-specific duplication events in these families, two or more genes from one species clustered together in a clade (bootstrap values > 50) were marked as species-specific duplication events in the seven NJ trees (Additional file 5: Fig. S1). There were 9, 8, 3, 5, 7, 3, and 1 species-specific duplication events involving 15, 15, 6, 10, 11, 6, and 2 genes in family679, family730, family1336, family2291, family4459, family4952, and family5347, respectively (Additional file 5: Fig. S1). The results demonstrated that 65 genes (65/156 = 41.67%) were involved in species-specific duplications among the seven young duplicate gene families.

### Duplication types of the young duplicate genes

The young duplicate genes could be classified into three duplication types, containing tandem duplication, transposon-related duplication and WGD, at the genome-wide level among the six Rosaceae species. In species-specific and lineage-specific expansions, young duplicate genes were involved in all the three duplication modes, but distinct gene numbers and percentages were displayed in different duplication types in the six species (Table 2). For example, there were relatively lower gene numbers and proportions in the three duplications types among species-specific and lineage-specific expansions in *F. vesca* and *R. occidentalis.*

**Table 2:** Gene numbers and percentages of young duplicate genes from three duplication types in species-specific and lineage-specific expansions

Species		Species-specific expansions				Lineage-specific expansions			
		Tandem Duplication	Transposed Duplication	Whole Genome Duplication	Total number	Tandem Duplication	Transposed Duplication	Whole Genome Duplication	Total number
***F. vesca***	**Number**	217	158	83	2932	148	118	91	1092
	**Percentage**	7.40%	5.39%	2.83%		13.55%	10.81%	8.33%	
***M. x domestica***	**Number**	3247	3044	2874	15,141	2765	3680	7287	18,221
	**Percentage**	21.45%	20.10%	18.98%		15.17%	20.20%	39.99%	
***P. communis***	**Number**	863	1332	1937	6885	1693	2631	6292	14,028
	**Percentage**	12.53%	19.35%	28.13%		12.07%	18.76%	44.85%	
***P. persica***	**Number**	1244	158	154	3314	130	444	522	2428
	**Percentage**	37.54%	4.77%	4.65%		5.35%	18.29%	21.50%	
***R. chinensis***	**Number**	1017	409	532	6804	233	170	187	1806
	**Percentage**	14.95%	6.01%	7.82%		12.90%	9.41%	10.35%	
***R. occidentalis***	**Number**	173	90	36	1734	55	93	64	968
	**Percentage**	9.98%	5.19%	2.08%		5.68%	9.61%	6.61%	

Number means number of the young duplicate genes from different duplication types in the two patterns of expansions in every species

Percentage means the gene number of each duplication type/the total gene number of species-specific expansion in each species or the gene number of different duplication types/ the total gene number of lineage-specific expansion in each species

Total number represents the total gene number of young duplicate gene of species-specific expansion in each species or the total gene number of young duplicate gene of lineage-specific expansion in each species

In species-specific expansions, the gene numbers of tandem duplications were much higher than those of the other two duplication types in *F. vesca*, *M. x domestica*, *P. persica*, *R. chinensis* and *R. occidentalis.* Accordingly, the highest percentages of the young duplicate genes came from tandem duplications were also detected in the five species. It was indicated that tandem duplications played important roles in the young duplications after the speciation of the five plants*.* Especially, 37.54% of the young duplicate genes were produced by tandem duplications in *P. persica*, representing the highest percentage compared with the proportions of this duplication type in the other species. However, in *P. communis*, the largest gene number and proportion were discovered in WGDs.

In lineage-specific expansions, young duplicate genes partly changed the distributions in the three duplication types compared with those of species-specific expansions. The largest gene numbers were detected in tandem duplications of *F. vesca* and *R. chinensis*, and their related proportions were 13.55 and 12.90% in the two species, respectively. More young duplicate genes were derived from WGDs in *M. x domestica*, *P. communis* and *P. persica*, and from transposon-related duplications in *R. occidentalis*. It is worth noting that relatively large percentages of young duplications were belonging to the WGDs in *M. x domestica* (39.99%) and *P. communis* (44.85%), but lower percentages of tandem duplicated genes in the two species (15.17% in *M. x domestica* and 12.07% in *P. communis*). The results illustrated WGDs driven the expansions of young duplicate genes in *M. x domestica*, *P. communis* and *P. persica* before the species differentiation and divergence. Therefore, all of these demonstrated that tandem duplications and WGDs might be the major force promoting the occurrence of young duplicate genes in the six Rosaceae species.

### Domain preference of the young duplicate genes

The protein domains of the young duplicates were explored in the species-specific and lineage-specific expansions to uncover the functional preference of the duplicate genes among the six Rosaceae species.

**Fig. 1:**
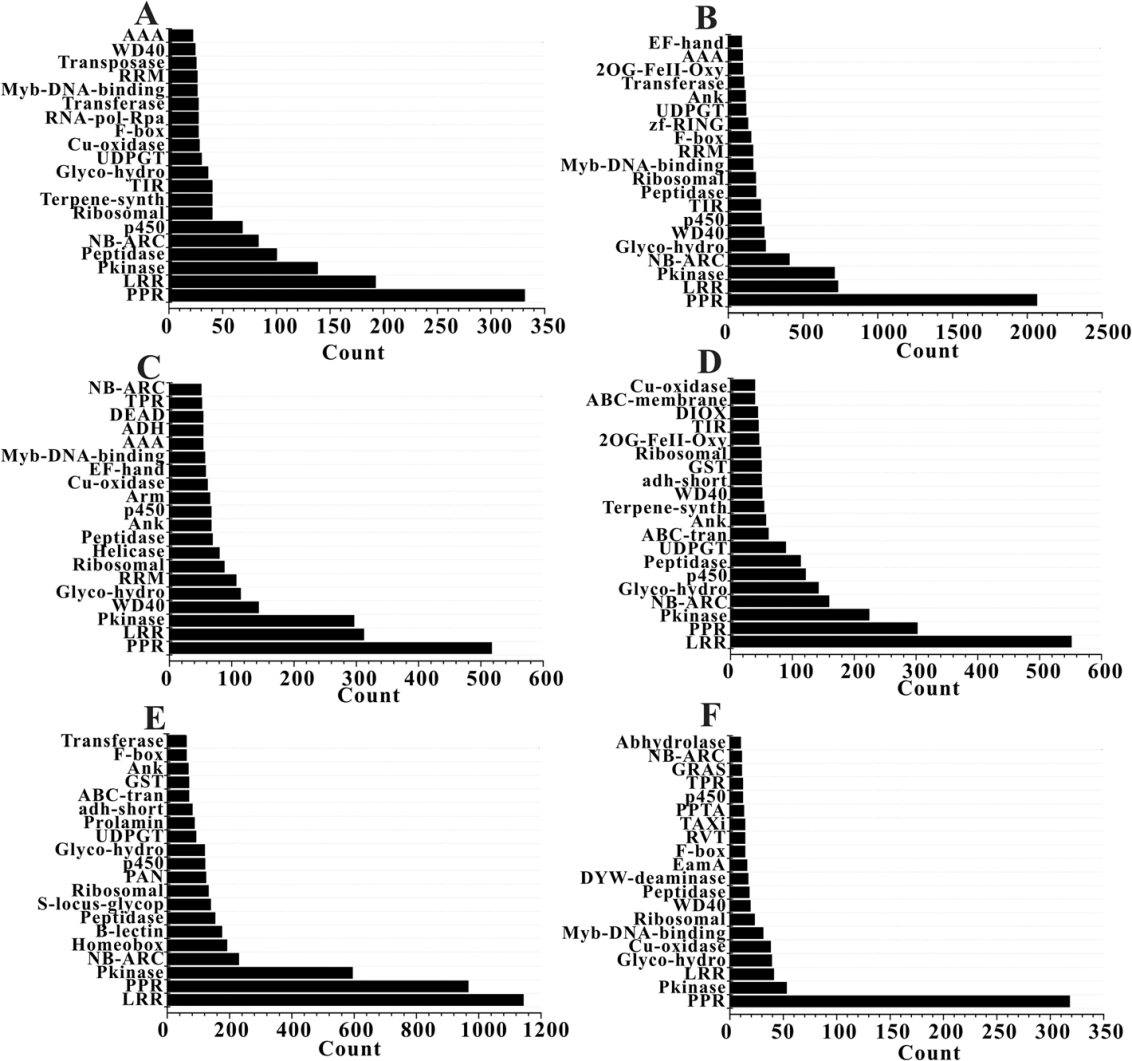
Top 20 protein domains of the young duplicate genes in species-specific expansions. The x-axis means the numbers of different domains. The y-axis means the domains taking the top 20 places of domain numbers. a: *F. vesca*, **b**: *M. x domestica*, **c**: *P. communis*, **d**: *P. persica*, **e**: *R. chinensis* and **f**: *R. occidentalis*

A total of 2117 different domains were detected in the species-specific expansions among the six species (Additional file 2: Table S2). It is worth mentioning that 43.50% (921/2117) of the domains appeared in only one species, indicating that approximately one half of the protein domains were uniquely encoded by species-specific duplicate genes in the six species. On the contrary, only 5.15% (109/2117) of the domains occurred simultaneously in all the six species. Interestingly, the low-frequency domains were relatively low in number in all the species, while some of the high-frequency domains were high in number in the related species (Fig. 1). For example, there were many high-count domains, especially the domains of PPR, LRR, Pkinase, p450, and NB-ARC, shared by the species-specific duplicates of the six Rosaceae species.

**Fig. 2:**
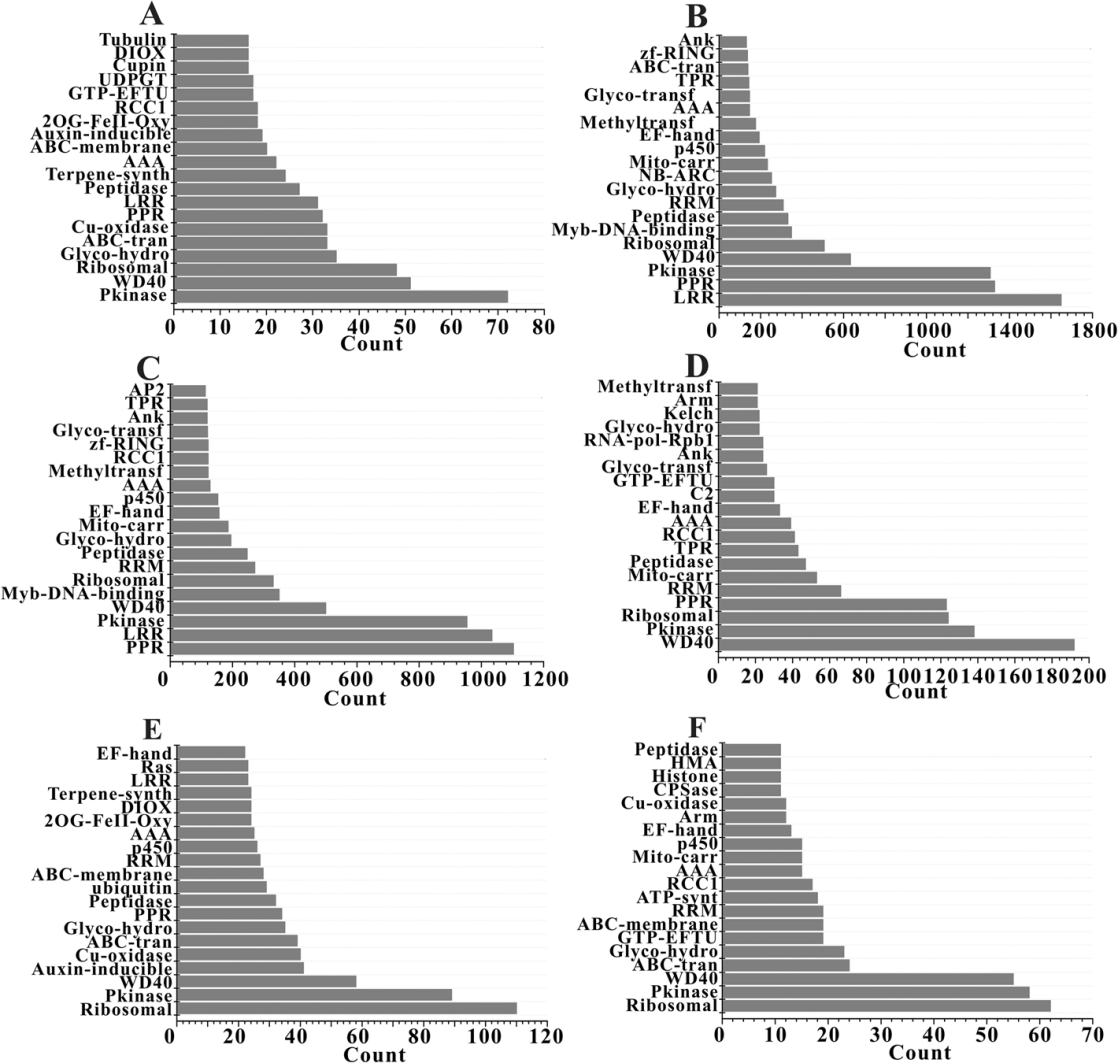
Top 20 protein domains of the young duplicate genes in lineage-specific expansions. The x-axis means the numbers of different domains. The y-axis means the domains taking the top 20 places of domain numbers. **a**: *F. vesca*, **b**: *M. x domestica*, **c**: *P. communis*, **d**: *P. persica*, **e**: *R. chinensis* and **f**: *R. occidentalis*

Although the numbers of domains found in lineage-specific expansions (2000) and in species-specific expansions were more or less equal, the domain frequency detected in both type of expansions was distinctly different. Clearly, only 5.20% of the protein domains (104/2000) were discovered in one species, such as B-lectin, Vicilin, and Trigger, demonstrating that a small amount of lineage-specific duplicate genes had exclusive domains in some species (Additional file 2: Table S2). In addition, 22.95% of the protein domains (459/2000) were found to co-occur in all the six species, with 7.56% (151/2000), 4.85% (97/2000), 25.30% (506/2000), and 34.15% (683/2000) of them appearing simultaneously in the lineages of five, four, three, and two species, respectively. Similar to the high-frequency domains of species-specific expansions, the domains of lineage-specific expansions also exhibited high occurrence in all the six species and also possessed a large number of copies in them, containing the Pkinase, PPR, LRR, p450, WD40, and Ribosomal, etc. (Fig. 2). Therefore, it may be concluded that the high-frequency duplicate domains in species-specific and lineage-specific expansions, involved in growth and development (Ribosomal, Ank, and Peptidase) or response to environmental stresses (PPR, NB-ARC, LRR, and Pkinase), might play a key role in the evolutionary processes of the six Rosaceae species.

### Duplication time of the young duplicate genes

The *Ks* values are molecular scales of duplication time and the divergence time. To further detect the timing of young duplication events in the six Rosaceae species, *Ks* values were calculated in both species-specific and lineage-specific duplicate gene families.

**Fig. 3:**
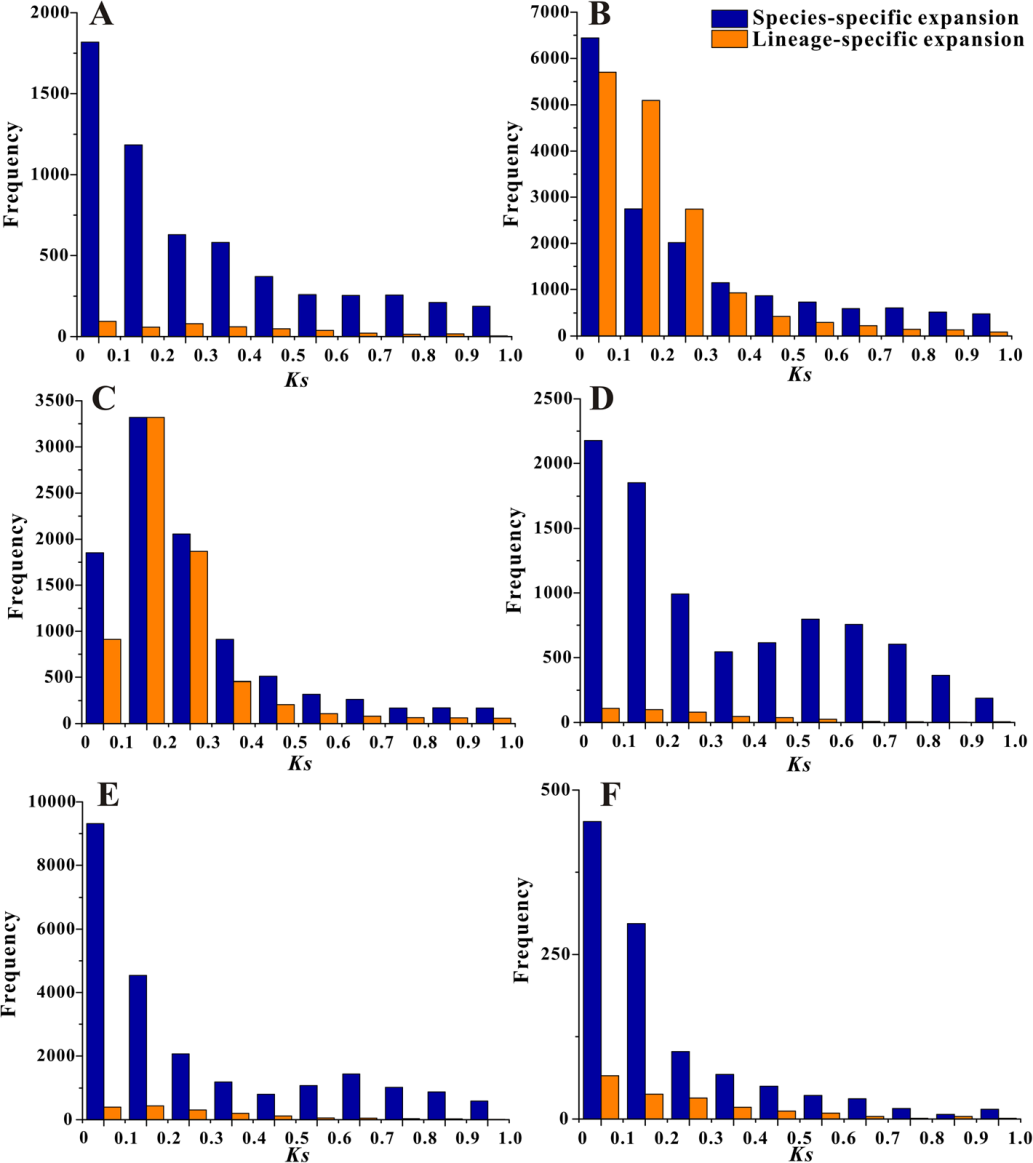
The *Ks* values of paralogs of young duplicate gene families in the two types of expansions. The x-axis means the range of *Ks* values from 0 to 1, and the range was divided into ten parts in unit of 0.1. The y-axis represents the occurrence of *Ks* value in each unit. **a**: *F. vesca*, **b**: *M. x domestica*, **c**: *P. communis*, **d**: *P. persica*, **e**: *R. chinensis* and **f**: *R. occidentalis*

In species-specific expansions, the average *Ks* values of the orthologs were higher than those of the paralogs only in *P. communis*, *R. chinensis* and *R. occidentalis* (Table 3). However, the *Ks* values of paralogs obviously peaked at the range of 0 to 0.1 with extremely high frequency and slowly decreased from 0.1 to 1 in all species, except *P. communis*, in which the *Ks* values peaked at the range from 0.1 to 0.2 (Fig. 3). These results illustrated that a considerable portion of the young duplicate genes were generated at the very recent times. In the lineage-specific expansions, the orthologs had larger *Ks* values than paralogs, which suggested that species divergence was followed by duplication events. In addition, the *Ks* values distributed differently with lower frequency from 0 to 1 compared with those in species-specific expansions. For example, the peak values of *Ks* were in the range of 0–0.1 in *F. vesca*, *M. x domestica*, *P. persica* and *R. occidentalis*, 0.1 to 0.2 in *P. communis* and *R. chinensis* (Fig. 3). Although the peaks were still detected at 0 to 0.1 in four species, no extreme advantage in *Ks* frequency compared with those at the range of 0.1–0.2 or 0.2–0.3. The observation proved that, in the period of the recent time, much more species-specific duplicate genes were produced than the lineage-specific ones. Moreover, the appreciable clustering of the *Ks* values around 0.2 in *M. x domestica* and *P. communis* of lineage-specific expansions was consistent with the recent WGD in the two species [21, 22].

**Table 3:** Average *Ks* values and *Pi* values of young duplicate gene families for two types of expansions

Species	Species-specific expansions				Lineage-specific expansions			
	Paralogs		Orthologs		Paralogs		Orthologs	
	***Ks***	***Pi***	***Ks***	***Pi***	***Ks***	***Pi***	***Ks***	***Pi***
***F. vesca***	0.28	0.32	0.28	0.18	0.32	0.21	0.47	0.26
***M. x domestica***	0.25	0.25	0.25	0.16	0.17	0.16	0.26	0.18
***P. communis***	0.25	0.28	0.28	0.21	0.21	0.18	0.25	0.17
***P. persica***	0.33	0.48	0.33	0.12	0.25	0.14	0.37	0.16
***R. chinensis***	0.26	0.28	0.30	0.20	0.25	0.15	0.46	0.25
***R. occidentalis***	0.19	0.26	0.27	0.19	0.22	0.16	0.41	0.21

### The nucleotide diversity of the young duplicate genes

To deeper explore the evolutionary differences between paralogs and orthologs, we calculated the nucleotide diversity values (*Pi* values) among species-specific and lineage-specific duplicate genes (Table 3).

In species-specific expansions, the paralogs had larger average *Pi* values than the orthologs in each of the six species. Moreover, *t*-test analysis were also operated between the *Pi* values of paralogs and orthologs, showing *Pi* values of paralogs were significantly higher than those of orthologs in each of the six species (*P* < 0.01). The results manifested that copies derived from species-specific duplications (paralogs) might undergo a relative faster sequence divergence leading to the larger diversities among paralogs than orthologs in the six species.

However, the opposite results of paralogs with lower average *Pi* values than the orthologs were found in lineage-specific expansions of the studies species, except *P. communis*. The paralogs have significantly smaller *Pi* values than the orthologs in *F. vesca*, *M. x domestica*, *P. persica*, *R. chinensis* and *R. occidentalis* (*t*-test, *P* < 0.01). It could be inferred that the ancestor copies inherited from ancestor species to the studies species (orthologs) might be driven by a faster divergence speed after the lineage-specific duplications in the five species.

### Selective pressure on young duplicate genes

The ratio of nonsynonymous to synonymous substitution (*Ka*/*Ks*) is an important indicator of the functional constraints on genes. Therefore, the *Ka*/*Ks* ratios of paralogs and orthologs were examined in all species-specific and lineage-specific duplicate gene families.

**Fig. 4:**
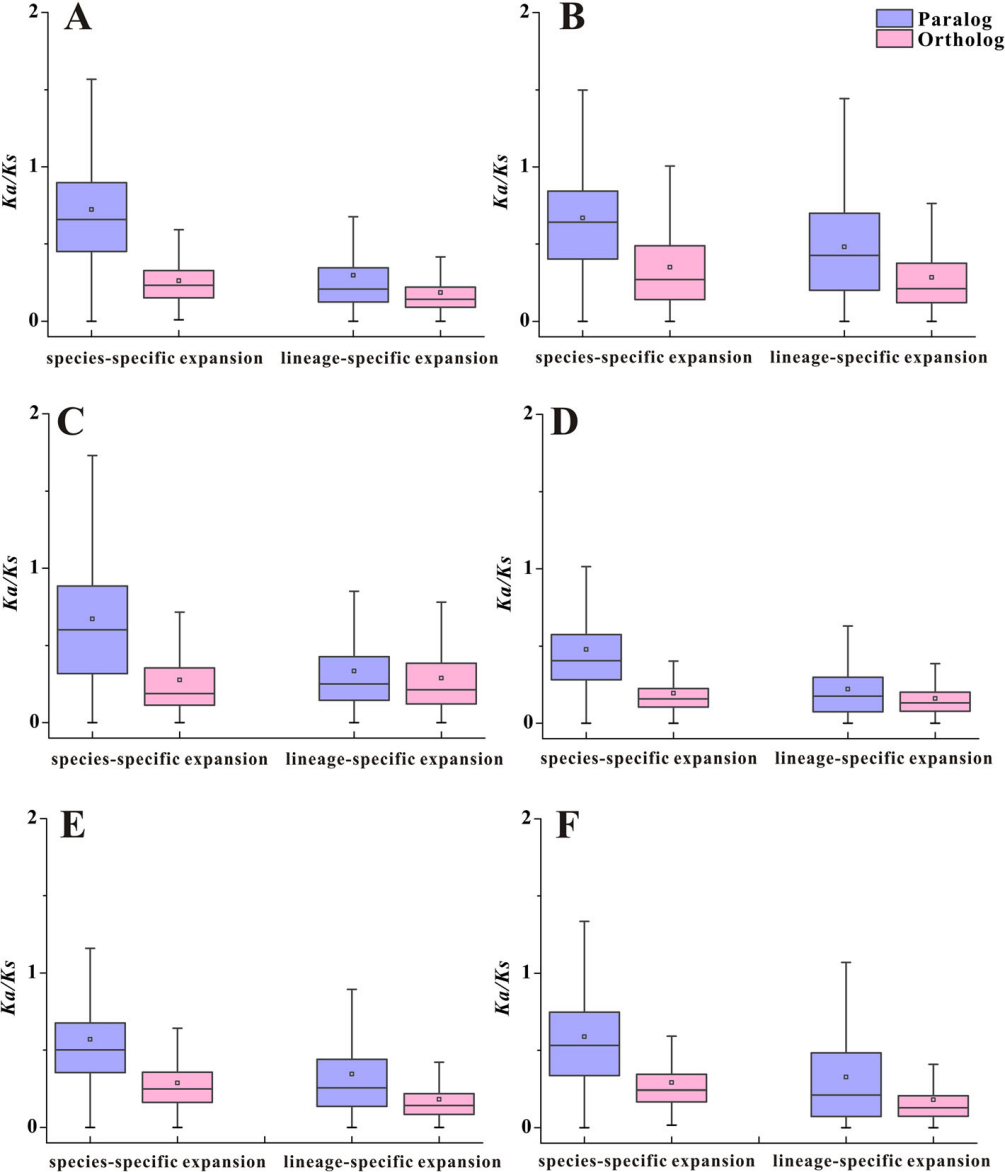
The *Ka*/*Ks* ratios of young duplicate genes in the two types of expansions. The box plots are exhibiting the distributions of *Ka*/*Ks* values among paralogs and orthologs in the two expansion types. The small square and the line in the box represent average and median values of the *Ka*/*Ks* values, respectively. **a**: *F. vesca*, **b**: *M. x domestica*, **c**: *P. communis*, **d**: *P. persica*, **e**: *R. chinensis* and **f**: *R. occidentalis*

In both species-specific and lineage-specific expansions, most of the gene pairs with *Ka*/*Ks* ratios smaller than 1 illustrated that a majority of the young duplicate genes were subject to purifying selection among the six Rosaceae species. Nevertheless, a fraction of the gene pairs showed *Ka*/*Ks* ratios greater than 1, suggesting that they underwent positive selection (Fig. 4). In species-specific expansions, the paralogs had greater median and average values compared with the orthologs in the six Rosaceae species. Moreover, the *Ka*/*Ks* ratios exhibited highly significant differences between paralogs and orthologs in each species (*t*-test, *P* < 0.01), demonstrating that paralogs had significantly larger *Ka*/*Ks* values than orthologs in species-specific young duplicate gene families among the six Rosaceae species. A similar phenomenon was observed in lineage-specific expansions, the paralogs had highly significantly greater *Ka*/*Ks* values than the orthologs in all the six species (*t*-test, *P* < 0.01). These results indicated that paralogs were driven by weaker functional constraints and had faster evolutionary rates than orthologs in the young duplicate gene families of the six Rosaceae species.

Furthermore, the phenomena were more directly displayed by the linear analysis of *Ka*/*Ks* ratios between paralogs and orthologs from the same young duplicate family in species-specific and lineage-specific expansions (Additional file 6: Fig. S2). The paralogs had higher *Ka*/*Ks* values than the orthologs of the same family and are represented by the corresponding dots above the trend lines (blue lines: slope equal to 1). Therefore, the farther the dots were from the trend lines, the faster did the evolutionary rates occur in the related family. The protein domains of these families were examined, and it was found that some of them were connected with in response to biotic or abiotic stresses, including PPR, FAR1 and UDPGT (Additional file 3: Table S3).

### Chromosomal location of young duplicate genes

The physical location of the young duplicate genes, in both the species-specific and lineage-specific expansions, was uneven on the chromosomes in the six Rosaceae species. Accordingly, the trends of the gene densities were basically consistent with those of gene numbers in the six species.

In species-specific expansions, four distribution patterns of the duplicate genes on the chromosomes in the six species were noticed (Additional files 7, 8, 9, 10, 11 and 12: Figs. S3–S8). In the first pattern, more duplicate genes preferred to distribute themselves in the regions near the two telomeres on each chromosome, such as in chromosomes 3, 5, and 6 of *F. vesca* and chromosomes 2, 3, 7, 8, 9, 10, 11, 14, 15, and 17 of *M. x domestica*. In the second pattern, the species-specific duplicate genes exhibited peak distributions in the neighborhood of one of the telomeres on each chromosome, such as in chromosomes 1, 4, 5, 6, 13, and 16 of *M. x domestica*. In the third pattern, there was a relative mean distribution of the young duplicate genes on the chromosomes, such as on all chromosomes of *P. communis* and *R. occidentalis*, chromosomes 3, 4, 5, and 6 of *P. persica*, and chromosomes 2, 4, and 5 of *R. chinensis*. In the fourth pattern, the peaks of the duplicate genes were located on the non-telomere regions of the chromosomes, such as in chromosomes 1 and 2 of *P. persica* and chromosomes 1, 6, and 7 of *R. chinensis*. Most of the chromosomes belonged to the first, third and forth patterns showed the gene densities consistent with the distribution pattern of the young duplicate numbers. That is, the chromosomal regions with gene number peaks generally presented gene density peaks, while the non-peak regions were also shared by the similar distributions between the gene numbers and gene densities, especially the related chromosomes from *F. vesca* and *R. chinensis*. Although similar distribution trends were revealed between the gene numbers and gene densities, there were some differences at some positions of the chromosomes. For instance, no number peak on the right telomere region of chromosome 4 from *P. persica*, but a density peak of young duplicate genes displayed here. This result demonstrated relatively lower genes distributed at this telomere region. The similar phenomenon was also exhibited in chromosome 1 of *R. occidentalis*.

Similarly, the four distribution patterns of the duplicate genes were also detected in lineage-specific expansions (Additional files 7, 8, 9, 10, 11 and 12: Figs. S3–S8). In the first pattern, a clear clustering around the two telomeres was found in chromosomes 2, 3, 7, 8, 9, 10, 11, 14, 15, and 17 of *M. x domestica* and chromosomes 3 and 6 of *P. persica*. In the second pattern, it was demonstrated by chromosomes 1, 4, 5, 6, 13, and 16 in *M. x domestica* and chromosomes 2, 4, 5, 7, and 16 in *P. persica*. The distribution tendency of the third pattern was revealed by all the chromosomes of *F. vesca*, *P. communis,* and *R. occidentalis*, and chromosomes 1, 2, 3, and 6 of *R. chinensis*. The fourth pattern was represented by chromosome 8 in *P. persica* and chromosomes 4, 5, and 7 in *R. chinensis*. Moreover, the positions with lower gene numbers but higher gene densities was also discovered in different chromosomes, such as chromosome 6 in *M. x domestica* and chromosome 12, 15 of *P. communis*. Interestingly, for the chromosomal locations of young duplicates in lineage-specific expansions, the extremely similar distributions between the gene numbers and gene densities were also discovered in all the chromosomes of *F. vesca* and *R. chinensis*. Therefore, the results indicated that the distribution patterns of young duplicate genes on the same chromosomes between the species-specific and lineage-specific expansions were similar on some of the chromosomes among the six species.

To further verify the relationship of gene locations between the two expansion types, we calculated the correlation coefficients of the young duplicate gene numbers between species-specific and lineage-specific expansions on the same chromosome in the six species (Additional file 4: Table S4). Most of the correlation coefficients of *M. x domestica* and some of them of *P. communis* were greater than 0.6, which indicated a strong correlation between the locations of species-specific and lineage-specific duplicate genes in the corresponding chromosomes.

### Collinearity between young duplicate genes

For exploring the evolutionary history of the young duplicate genes, the collinearity block analysis was performed among the young duplicate genes of lineage-specific expansions co-occurred in the six species. The family members and their neighbouring genes predicted in the collinearity blocks were showed across the corresponding chromosomal regions (Additional file 13: Fig. S9). Although the collinearity blocks located in different chromosomes among the six species, the young duplicate genes of the four families with collinearity relationships were frequently found distributed in the chromosome 3 in *F. vesca*, chromosome 3, 5, 10 and 11 in *M. x domestica* and *P. communis*, chromosome 4 and 6 in *P. persica*, chromosome 5 in *R. chinensis*, and chromosome 3 in *R. occidentalis*, respectively. These indicated that these chromosomal regions might originate from the ancestral chromosomes of the common ancestor species of Rosaceae. In family679, one *F. vesca* member, two *M. x domestica* members, one *P. communis* member, one *P. persica* member, one *R. chinensis* member, and one *R. occidentalis* member were predicted locating in the same collinearity block, and the other shared collinearity block contained one, two, two, one, one and one members from *F. vesca*, *M. x domestica*, *P. communis*, *P. persica*, *R. chinensis*, and *R. occidentalis*. Similarly, there were also one or two members from each species involved in the same collinearity block in the other three families. Interestingly, some members and their neighbouring genes still represented the consistent transcriptional directions after the long evolutionary processes, such as the members from *F. vesca* (Fv3g35380) and *R. chinensis* (RC5G0545100) in familiy679. The family members along with the related neighbouring genes exhibited these ancestral regions, which were conserved and retained in the chromosomes of the six modern Rosaceae species after experiencing series of evolutionary events, including the young duplications.

## Discussion

### Rosaceae evolution history exerting influences on the young duplicate genes

To retrospect the history of the Rosaceae family, it could be better discovered the characteristics of young duplicate genes reflected by the evolutionary processes of the six species.

In the ancestor genome of Rosaceae family, nine protochromosomes represent the ancestral Rosacese karyotype, which experienced varying scales of fusions and fissions at chromosome level to successfully develop into the ancestral karyotype of subfamilies, such as ancestral *Rosoideae* karyotype and ancestral *Prunoideae* karyotype [19]. There are different evolutionary processes happen in ancestral *Maloideae* karyotype compared with the former two karyotypes, that is, duplications and large scales of chromosome fusions and fissions contribute to the generating of ancestral *Maloideae* one [19]. A recent WGD shared by the modern Rosaceae species of *Malus* and *Pyrus* is also reported, which might be one of the main factor leading to the large numbers of young duplicate families in the lineage of *M. x domestica* and *P. communis* (Table 1) [21, 22, 26]. In addition, this recent WGD might also explain the result, for which the young duplicate genes belonging to WGDs accounted for larger proportions in *M. x domestica* and *P. communis* from lineage-specific expansions (Table 2). On the contrary, the other four Rosaceae genomes lack the similar duplication observed in *Malus* and *Pyrus* species, but some fragmentary triplicated sub-genomic blocks are in *P. persica* genome [19, 20, 23, 25]. Therefore, these phenomena supported the relatively lower young duplicates and lower numbers of WGDs in *F. vesca*, *R. chinensis* and *R. occidentalis*, and a little higher number of WGDs in *P. persica* from lineage-specific expansions (Table 2). Besides, the duplication time of the young duplicate genes in *M. x domestica* and *P. communis* from the lineage-specific expansions (Fig. 3) partly coincided with the shared recent WGD in *Malus* and *Pyrus* species (*Ks* peak around 0.2) [21]. These demonstrated that the recent WGD might partly contribute to the generating of lineage-specific young duplicate genes in the two species.

Although different evolutionary pathways shaped distinct modern Rosaceae genomes, some shared ancestral regions/genes were still detected in their existing chromosomes among the six Rosaceae species (Fig. S9). These further indicated the studied species originated from the common ancestor, and still retained some ancestral genetic traits in their modern genomes [27].

### Functional preference of young duplicates adaptation to environmental stresses

Duplicated genes were previously represented in response to different ecological conditions in various plants [1]. For example, the gene duplicates were adaptation to extreme temperature in *Arabidopsis* and Brassicaceae, salt stress in *Citrus* and *Sorghum bicolor*, drought in wild tomato, weedicides in *Medicago sativa*, *Glycine max* and *Nicotiana tabacum*, nutrient limitation in Poaceae, and pathogen infections in *Arabidopsis* and *O. sativa* [28–34]. In our study, the domains of PPR, NB-ARC, LRR, Pkianse, Ribosomal, Ank and Peptidase exhibited high frequency in species-specific and lineage-specific duplications, which were involved in the adaptation to the changing environmental forces in plants.

The pentatricopeptide repeat (PPR) protein is composed of the PPR motif, a degenerate 35-amino-acid repeat tandemly arranged for 2 to 26 copies [35]. In plants, the PPR gene family is one of the largest gene families because of its extreme expansions in copy number, which makes it broadly function as gene-specific transcriptional regulators in the mitochondria and chloroplasts in response to ecological factors [36]. A mitochondrial PPR protein, PPR40, has created a close connection between oxidative respiration and stress/hormonal responses in *A. thaliana* [37]. In addition, some of the *PPR* genes, such as the restorer-like genes, are subject to diversifying selection, associating with the “arm-race” co-evolution of nuclear and cytoplasmic genomes in plants [38–40]. The *PPR* genes going through duplication and divergence have some characteristics similar to those of *R* genes, and they may play a vital role in adaptive evolution by the modification of cytoplasmic proteins in plants [39, 40].

The *NBS-LRR* gene family is one of the largest disease resistance gene families in plants, which encodes the NBS-LRR proteins mainly composed of the NB-ARC and LRR domains [41]. Plant NBS-LRR proteins, ancient in origin and abundant in copy numbers, are involved in the broad-spectrum resistance against various pathogens including fungi, viruses, bacteria, oomycetes, insects, and nematodes [41, 42]. The presence of a *Pi2/9* locus containing tandemly arranged *NBS-LRR* genes with a variety of copy numbers and high levels of divergence in the rice genome suggests that the genes of this locus adaptively cope with diverse pathogens [43]. In the Pi2/9 locus, genes *Pi2* and *Pi9* exhibit broad-spectrum resistance to rice blast [44, 45]. Similarly, many of the plant *NBS-LRR* genes are in the phase of developing a rapid evolutionary pattern driven by diversifying selection, which is accomplished by numerous duplications, frequent sequence exchanges, and pseudogene losses [46]. These evolutionary processes facilitate the fast divergence of *NBS-LRR* genes to adapt to the rapidly changing pathogens [46, 47].

The protein kinase (*Pkinase*) family is one of the largest superfamilies in eukaryotic organisms, composed of a mass of homologous proteins with common conserved structural motifs [48]. However, the members of the Pkinase family exhibited an abundant variation in their structures, regulation patterns, and substrate specificities and functions involved in many cellular processes such as development, differentiation, proliferation, and defense responses against abiotic or biotic stresses [48, 49]. Especially, many of the leucine-rich repeat receptor-like kinase (*LRR-RLK*) genes are part of the disease resistance gene family in plants [16]. The *LRR-RLK* genes involved in interactions with the environment are mainly detected in lineage-specific duplicate clades driven by positive selection in plant genomes [50, 51]. For example, an *LRR-RLK* gene, *Xa21*, from wild rice, *O. longistaminata*, showed broad-spectrum resistance to bacterial blight (*Xanthomonas oryzae* pv. *oryzae*) [52, 53]. Among the *Xa21* homologous loci in gramineous genomes, the frequent occurrence of duplication, gene translocation, and copy number variation demonstrated that *Xa21* homologs undergo rich diversification in order to adapt to the specific fast-changing pathogens over long evolutionary timescales [54].

### Adaptive evolution of young duplicate genes

Gene families are driven by adaptive evolution following gene duplications, which is represented by duplicate genes under positive selection [1, 55]. In this study, a part of gene pairs from species-specific and lineage-specific expansions with *Ka*/*Ks* values greater than 1 indicated that the genes were driven by adaptive evolution in the six Rosaceae species. For example, NBS-LRR genes, a large family of disease resistance genes, always experience large-scale gene duplications and rapid adaptive evolution in many plant genomes, including several Rosaceae species [16, 17]. Based on the divergence analysis of the *Ka*/*Ks* (or *dN*/*dS*) ratios, it has been previously reported that many gene families in plants are driven by adaptive evolution. For example, the monosaccharide transporter (*MST*) gene family, an ancient origin and largely duplicate family, reveals evidence of positive selection in rice and *Arabidopsis* [56]; the chalcone synthase (*CHS*) multigene family, which plays an important role in flavonoid biosynthesis and the divergence of its three subfamilies (SF1, SF2, and SF3), has been under positive selective pressure in the *Dendranthema* species [14]; and the S-phase kinase-associated protein 1-like (Skp1-like) and F-box families also show a similar pattern of adaptive evolution in *A. thaliana* [57, 58].

Moreover, paralogs had faster evolutionary rates than orthologs with *Ka*/*Ks* ratios greater than 1 among the young duplicate gene families, which could be inferred based on the fact that the former might undergo a higher frequency of adaptive evolution than the latter in the six Rosaceae species [59]. Instances contain genes involved in response to ecological conditions of different stresses under adaptive evolution among the species-specific duplicate families, including *LEA* genes in *M. x domestica*, FAR-RED IMPAIRED RESPONSE1 (*FAR1*) genes in *P. communis*, and the glucuronyltransferase family (UDPGT domain, *UGT*) in *R. chinensis* (Additional file 3: Table S3). In our study, species-specific expansions involved more gene members and gene families compared with lineage-specific expansions among the six Rosaceae species. The results demonstrated that the species-specific duplications mainly contributed to the recent expansions of the six species. It was previously reported that species-specific duplications could result in species-specific functions and traits in a given species, helping the species to maintain better growth and development and exploit stronger adaptability to the constantly changing environment [16, 60].

In apple trees, a period of exposure to low temperature was necessary to enable dormancy induction and growth resumption [61]. The *LEA* genes encode late embryogenesis abundant proteins in higher plants, many of which are seen to play a role in environmental stress response, such as drought, high salinity, and low temperature [62–64]. For example, over-expression of the *OsLEA3–2* gene can enhance the salt and drought tolerance of transgenic *Arabidopsis* and rice during growth periods [65]. In woody plants, including apples, peaches, and grapes, the *LEA* genes are the major ones among the consistently up-regulated genes induced by the conditions of photoperiod and low temperature [66]. Therefore, it may be inferred that the apple-specific duplication of *LEA* genes under positive selection might be closely associated with adaption to the physiological characteristic of dormancy.

Species-specific duplications of *FAR1* were detected with the feature of adaptive evolution in the pear genome, which are photophilous fruit trees requiring approximately 1600–1700 h of sunlight annually. *FAR1* and FAR-RED ELONGATED HYPOCOTYL3 (*FHY3*) are light-signaling transcription factors, which regulate light-induced inositol biosynthesis, oxidative stress response, starch synthesis, and starch granule formation [67, 68]. The FAR1 and FHY3 proteins promote the tolerance to oxidative stress and prevent the salicylic-acid-dependent cell death by activating the myo-inositol-1-phosphate synthase1 gene (*MIPS1*) to adapt to the light changes in *Arabidopsis* [67]; they also modulate light-induced starch synthesis by transcriptional activation of the starch-debranching enzyme ISOAMYLASE2 (*ISA2*) in response to light and sugars in *A. thaliana* [68]. Thus, we speculated that pears generate more *FAR1* genes to adapt to a given light environment for more sugar synthesis and accumulation for fruit ripening. Besides, half of the *FAR1* genes reveal up-regulation during salinity stress treatments in *Aegilops tauschii*, suggesting that they may play certain roles in salt stress response [69]. Pears have a relatively strong adaptability to ecological conditions, containing the tolerance to cold, drought, and salt stresses [70], indicating that the evolution of *FAR1* genes might support the species to adapt to different ecological conditions.

The *UGT* genes comprise the largest gene family in plants, responding to a variety of biotic and abiotic stresses by glycosylating various phytohormones and other metabolites [71, 72]. *UGT73B3* and *UGT73B5* in tomato and *AtSGT1* and UGT76B1 in *Arabidopsis* are involved in the biotic stress response to the infection caused by *Pseudomonas syringae* with the crosstalk of salicylic acid and jasmonic acid [73–75]. Similarly, the tobacco glycosyltransferase gene, *TOGT1*, plays an important role in enhancing the resistance to tobacco mosaic virus (TMV) [76]. In *Arabidopsis,* UGT genes, *UGT85U1/2* and *UGT85V1*, in response to abiotic stresses, are involved in improving the tolerance to salt and oxidative stress [77]. Therefore, all the above-mentioned examples suggest that duplications and adaptive evolution of *UGT* genes in Chinese rose (*R. chinensis*) might be driven by specific ecological conditions, including biotic or abiotic factors.

## Conclusions

In summary, this study uncovered the evolutionary patterns of young duplicate genes among the six Rosaceae species. All results showed that functional bias of the young duplicate genes was related to environmental stimuli response. The young duplicate genes were driven by adaptive evolution to adapt to the ecological factors in the six Rosaceae species. This work provides a systematic ways to study young duplications at genome-wide levels among closely related species.

## Methods

### Identification of young duplicate gene families

The whole genome sequences and annotations of the six Rosaceae species, *F. vesca* (v4.0.a1), *M. x domestica* (v1.0), *P. communis* (v1.0), *P. persica* (v2.0.a1), *R. chinensis* (v1.0) and *R. occidentalis* (v1.0), were downloaded from the Genome Database for Rosaceae (GDR, https://www.rosaceae.org/) [20–25, 78–80]. An all-vs.-all BLASTN search was performed among the nucleotide sequences (CDSs) of the six species (e-value< 1.0e-40) by using the local BLAST+ program. Based on the BLAST results, a PERL script was performed to identify gene families among the six species, for which the genes with coverage exceeded 60% were considered to constitute a gene family. Following this, these gene families that had at least two members from the same one species and those with the highest identity more than 90% were defined as young duplicate gene families by a PERL script [81].

Two types of expansions, species-specific and lineage-specific expansions, were detected across all young duplicate gene families. Species-specific expansion was characterized by young duplications occurring in only one species; that is, the corresponding species comprised ≥2 members and the other species comprised ≤1 member in each family. Lineage-specific expansion was characterized by the occurrence of young duplications in two or more species. That is, when the lineage-specific expansion was found in < 6 species (2 or 3 or 4 or 5), the involved species had ≥2 members and the other species had ≤1 member in each family; when the lineage-specific expansion was discovered in six species, all the studied species owned ≥2 members in each gene family.

### Identification of paralogs and orthologs

In each young duplicate gene family, their proteins sequences were used to run the script with default parameters by using OrthoFinder, and the members were classified into different orthogroups [82, 83]. In each orthogroup, genes derived from the same one species are defined as paralogs, and genes from different species are orthologs based on the illustration of OrthoFinder [82–84]. Taken a young duplicate family as an example, the family10 exhibiting the species-specific expansion occurred in *F. vesca* genome. There were four gene members in this family, two of them from *F. vesca* (Fv4g31010 and Fv4g31020), one from *R. chinensis* (RC7G0418500) and the other one from *R. occidentalis* (T00039). The four gene members were clustered in an orthogroup according to the Orthofinder identification. Therefore, the two genes from *F. vesca* were considered as paralogs; and the genes from different species, such as Fv4g31010 vs. RC7G0418500, were defined as orthologs. Similarly, the young duplicate families from the lineage-specific expansions also possessed the paralog and ortholog relationships between their gene members.

### Phylogenetic tree of young duplicate genes

For the accuracy of sequence alignments, CDSs of the seven young duplicate gene families involved in the six species were firstly translated into proteins, which experienced the alignments by using the MUSCLE program with default options in MEGA7 [85]. Then, the protein alignments were used to guide the alignments of nucleotide sequences, which were employed to constructed the phylogenetic trees using the neighbor-joining (NJ) method with 1000 replicates based on the p-distance model in MEGA7 [85].

### Duplication types of young duplicate genes

In each of the six species, the whole-genome protein sequences were considered as both database and query sequences to proceed a BLASTP search by the local BLAST+ program (e-value< 1.0e-40). Moreover, chromosomal locations of whole-genome genes were achieved from the annotation files of the six species. Based on the BLAST results and the physical positions, different modes of duplication gene pairs were classified by using the comparative genomic tool DupGen_finder [86]. Afterwards, the duplication types of young duplicate genes were screened out from the genome-wide classification results in each species.

### Domain preference of young duplicate genes

The protein sequences of all young duplicate genes were obtained by a PERL script, used to study the structural domains of them by the local Pfam database with e-value 1.0 (http://pfam.xfam.org/). Then, the domain numbers of the young duplicate genes were counted by Excel according to the Pfam results.

### Calculation of nonsynonymous and synonymous values of young duplicate genes

Likewise, the CDS alignments of each young duplicate orthogroup were got from the guidance of protein alignments using the ClustalW2.1 program [87]. The nonsynonymous substitutions (*Ka*), synonymous substitutions (*Ks*), and the ratio of the nonsynonymous to synonymous substitutions (*Ka*/*Ks*) were calculated using MEGA7 based on the CDS alignments in each orthogroup [85]. For *Ks* ratios, the values less than 1 were retained considering the saturation of nucleotide substitutions. Afterwards, the counting statistics of *Ks* values were performed in units of 0.1 in paralogs and orthologs among the two types of expansions by Excel, respectively. Finally, the bar graphs of *Ks* values were generated by Origin. For *Ka*/*Ks* ratios, the values between paralogs and orthologs were sorted by Excel and the graphs were also drawn by Origin.

### Calculation of nucleotide diversity values of young duplicate genes

Based on the alignments of young duplicates in each orthogroup, the nucleotide diversity values (*Pi*) were calculated by MEGA7 [85]. Afterwards, the *Pi* values of paralogs and orthologs were sorted by Excel.

### Chromosomal location of young duplicate genes

The physical positions of young duplicate genes and whole-genome genes were available from the annotation information of the six species. In all species, each chromosome of them was divided into a quantity of windows (1 Mb/1 Window), and the gene numbers and gene densities of species-specific and lineage-specific expansions were calculated in windows on corresponding chromosome of each species, respectively.

### Syntenic analysis of young duplicate genes between species

The syntenic relationships between the genes of the six species were detected at genome-wide levels by using TBtools [88] and MCScanX [89]. After obtaining the syntenic blocks between the studied species, the related young duplicate genes from the lineage-specific expansions were picked out to perform the visualization of microsyntenic relationships.

## Supplementary Information

**Additional file 1: Table S1.** Number of young duplicate gene families for two types of expansions. The number near lineage-specific expansion means the species numbers involved in lineage-specific expansions. The plus sign means corresponding species involved in the lineage-specific expansions.

**Additional file 2: Table S2.** Domain frequency and corresponding gene number of young duplicate families in species-specific and lineage-specific expansions.

**Additional file 3: Table S3.** Protein domains of families represented by the dots far away from the trend lines.

**Additional file 4: Table S4.** The correlation coefficients of the young duplicate gene numbers between species-specific and lineage-specific expansions on the same chromosome.

**Additional file 5: Figure S1.** Phylogenetic trees of seven young duplicate gene families from the lineages of all six species. A: family679, B: family730, C: family1336, D: family2291, E: family4459, F: family4952, and G: family5347. Red, green, yellow, blue, brown and purple circles represent genes from *F. vesca*, *M. x domestica*, *P. communis*, *P. persica*, *R. chinensis* and *R. occidentalis*, respectively. The clade with bootstrap values larger than 50 is considered to detect the species-specific duplication and lineage-specific duplication. Blue box means species-specific duplications.

**Additional file 6: Figure S2.** The linear analysis of *Ka*/*Ks* ratios between paralogs and orthologs in species-specific and lineage-specific expansions. A-F: the *Ka*/*Ks* values of species-specific expansions from *F. vesca*, *M. x domestica*, *P. communis*, *P. persica*, *R. chinensis* and *R. occidentalis*, respectively; G: the *Ka*/*Ks* values of lineage-specific expansions. The x-axis represents *Ka*/*Ks* values among orthologs and the y-axis means *Ka*/*Ks* values among paralogs. Black lines represent the trend line of dots and blue lines means trend lines with slope = 1.

**Additional file 7: Figure S3.** Chromosomal locations of young duplicate genes in the two types of expansions in *F. vesca*. Blue lines mean young duplicate genes from species-specific expansions, in which the solid ones mean gene numbers and the dotted ones are gene densities; and red lines represent young duplicate genes from lineage-specific expansions, in which the solid ones mean gene numbers and the dotted ones are gene densities. The x-axes represent the chromosomes, the left y-axes mean gene number and the right y-axes mean gene density.

**Additional file 8: Figure S4.** Chromosomal locations of young duplicate genes in the two types of expansions in *M. x domestica*. Blue lines mean young duplicate genes from species-specific expansions, in which the solid ones mean gene numbers and the dotted ones are gene densities; and red lines represent young duplicate genes from lineage-specific expansions, in which the solid ones mean gene numbers and the dotted ones are gene densities. The x-axes represent the chromosomes, the left y-axes mean gene number and the right y-axes mean gene density.

**Additional file 9: Figure S5.** Chromosomal locations of young duplicate genes in the two types of expansions in *P. communis*. Blue lines mean young duplicate genes from species-specific expansions, in which the solid ones mean gene numbers and the dotted ones are gene densities; and red lines represent young duplicate genes from lineage-specific expansions, in which the solid ones mean gene numbers and the dotted ones are gene densities. The x-axes represent the chromosomes, the left y-axes mean gene number and the right y-axes mean gene density.

**Additional file 10: Figure S6.** Chromosomal locations of young duplicate genes in the two types of expansions in *P. persica*. Blue lines mean young duplicate genes from species-specific expansions, in which the solid ones mean gene numbers and the dotted ones are gene densities; and red lines represent young duplicate genes from lineage-specific expansions, in which the solid ones mean gene numbers and the dotted ones are gene densities. The x-axes represent the chromosomes, the left y-axes mean gene number and the right y-axes mean gene density.

**Additional file 11: Figure S7.** Chromosomal locations of young duplicate genes in the two types of expansions in *R. chinensis*. Blue lines mean young duplicate genes from species-specific expansions, in which the solid ones mean gene numbers and the dotted ones are gene densities; and red lines represent young duplicate genes from lineage-specific expansions, in which the solid ones mean gene numbers and the dotted ones are gene densities. The x-axes represent the chromosomes, the left y-axes mean gene number and the right y-axes mean gene density.

**Additional file 12: Figure S8.** Chromosomal locations of young duplicate genes in the two types of expansions in *R. occidentalis*. Blue lines mean species-specific expansions, in which the solid ones mean gene numbers and the dotted ones are gene densities; and red lines represent lineage-specific expansions, in which the solid ones mean gene numbers and the dotted ones are gene densities. The x-axes represent the chromosomes, the left y-axes mean gene number and the right y-axes mean gene density.

**Additional file 13: Figure S9.** Microsynteny of young duplicate genes in four young duplicate gene families from the lineage-specific expansions co-occurring in six species. A: family679, B: family730, C: family2291 and D: family4952. Fv: *F. vesca*, Md: *M. x domestica*, Pc: *P. communis*, Pp: *P. persica*, Rc: *R. chinensis* and Ro: *R. occidentalis*. The black lines and triangles mean the chromosomal regions and the related genes on them. Orange triangles represent the gene members from the four families and the green ones are their neighbouring genes. The directions of the triangles mean the transcriptional directions of the genes. The dashed lines indicate the relative long distances on the chromosomes. The oranges lines linking the gene members represent the collinearity relationships between them.

## Data Availability

All data generated or analysed during this study are included in this published article and supplementary information files.

## Abbreviations

WGD: whole-genome duplication
NBS-LRR: nucleotide-binding site leucine-rich repeat;
PPR: pentatricopeptide repeat
Pkinase: protein kinase
LRR-RLK: leucine-rich repeat receptor-like kinase
CDS: Nucleotide coding sequences
CENP-C: centromere protein C
CHS: chalcone synthase
MST: monosaccharide transporter
Skp1-like: S-phase kinase-associated protein 1-like
FAR1: FAR-RED IMPAIRED RESPONSE1
FHY3: FAR-RED ELONGATED HYPOCOTYL3
UGT: glucuronyltransferase
MIPS1: myo-inositol-1-phosphate synthase1 gene
ISA2: ISOAMYLASE2.

## References

[1] Ohno S (1970). Evolution by gene duplication.

[2] Long M, Langley CH (1993). Natural selection and the origin of jingwei, a chimeric processed functional gene in Drosophila. Science.

[3] Moore RC, Purugganan MD (2005). The evolutionary dynamics of plant duplicate genes. Curr Opin Plant Biol.

[4] Yuan JQ, Wang JP, Yu JG, Meng FB, Zhao YH, Li J, Sun PC, Sun SR, Zhang ZK, Liu C (2019). Alignment of Rutaceae genomes reveals lower genome fractionation level than Eudicot genomes affected by extra Polyploidization. Front Plant Sci..

[5] Lynch M, Conery JS (2000). The evolutionary fate and consequences of duplicate genes. Science.

[6] Panchy N, Lehti-Shiu M, Shiu S-H (2016). Evolution of gene duplication in plants. Plant Physiol.

[7] Flagel LE, Wendel JF (2009). Gene duplication and evolutionary novelty in plants. New Phytol.

[8] Blanc G, Hokamp K, Wolfe KH (2003). A recent polyploidy superimposed on older large-scale duplications in the Arabidopsis genome. Genome Res.

[9] Paterson AH, Bowers JE, Chapman BA (2004). Ancient polyploidization predating divergence of the cereals, and its consequences for comparative genomics. Proc Natl Acad Sci U S A.

[10] Vandepoele K, Simillion C, Van de Peer Y (2003). Evidence that rice and other cereals are ancient aneuploids. Plant Cell.

[11] Rodriguez-Trelles F, Tarrio R, Ayala FJ (2003). Convergent neofunctionalization by positive Darwinian selection after ancient recurrent duplications of the xanthine dehydrogenase gene. P Natl Acad Sci USA.

[12] Spillane C, Schmid KJ, Laoueille-Duprat S, Pien S, Escobar-Restrepo JM, Baroux C, Gagliardini V, Page DR, Wolfe KH, Grossniklaus U (2007). Positive darwinian selection at the imprinted MEDEA locus in plants. Nature.

[13] Talbert PB, Bryson TD, Henikoff S (2004). Adaptive evolution of centromere proteins in plants and animals. J Biol.

[14] Yang J, Huang JX, Gu HY, Zhong Y, Yang ZH (2002). Duplication and adaptive evolution of the chalcone synthase genes of dendranthema (Asteraceae). Mol Biol Evol.

[15] Yang SH, Zhang XH, Yue JX, Tian DC, Chen JQ (2008). Recent duplications dominate NBS-encoding gene expansion in two woody species. Mol Gen Genomics.

[16] Zhong Y, Yin H, Sargent DJ, Malnoy M, Cheng ZM (2015). Species-specific duplications driving the recent expansion of NBS-LRR genes in five Rosaceae species. BMC Genomics..

[17] Jia YX, Yuan Y, Zhang YC, Yang SH, Zhang XH (2015). Extreme expansion of NBS-encoding genes in Rosaceae. BMC Genet..

[18] Zhang SD, Jin JJ, Chen SY, Chase MW, Soltis DE, Li HT, Yang JB, Li DZ, Yi TS (2017). Diversification of Rosaceae since the late cretaceous based on plastid phylogenomics. New Phytol.

[19] Raymond O, Gouzy J, Just J, Badouin H, Verdenaud M, Lemainque A, Vergne P, Moja S, Choisne N, Pont C (2018). The Rosa genome provides new insights into the domestication of modern roses. Nature Genetics.

[20] Shulaev V, Sargent DJ, Crowhurst RN, Mockler TC, Folkerts O, Delcher AL, Jaiswal P, Mockaitis K, Liston A, Mane SP (2011). The genome of woodland strawberry (Fragaria vesca). Nat Genet.

[21] Velasco R, Zharkikh A, Affourtit J, Dhingra A, Cestaro A, Kalyanaraman A, Fontana P, Bhatnagar SK, Troggio M, Pruss D (2010). The genome of the domesticated apple (Malus x domestica Borkh.). Nature Genetics.

[22] Chagne D, Crowhurst RN, Pindo M, Thrimawithana A, Deng C, Ireland H, Fiers M, Dzierzon H, Cestaro A, Fontana P, et al. The draft genome sequence of European pear (*Pyrus communis* L. 'Bartlett'). PLoS One. 2014;9(4):e92644. 10.1371/journal.pone.0092644 PMC3974708 24699266

[23] Verde I, Abbott AG, Scalabrin S, Jung S, Shu SQ, Marroni F, Zhebentyayeva T, Dettori MT, Grimwood J, Cattonaro F (2013). The high-quality draft genome of peach (Prunus persica) identifies unique patterns of genetic diversity, domestication and genome evolution. Nat Genet.

[24] Saint-Oyant LH, Ruttink T, Hamama L, Kirov I, Lakhwani D, Zhou NN, Bourke PM, Daccord N, Leus L, Schulz D (2018). A high-quality genome sequence of Rosa chinensis to elucidate ornamental traits. Nat Plants.

[25] VanBuren R, Bryant D, Bushakra JM, Vining KJ, Edger PP, Rowley ER, Priest HD, Michael TP, Lyons E, Filichkin SA (2016). The genome of black raspberry (Rubus occidentalis). Plant J.

[26] Wu J, Wang Z, Shi Z, Zhang S, Ming R, Zhu S, Khan MA, Tao S, Korban SS, Wang H (2013). The genome of the pear (Pyrus bretschneideri Rehd.). Genome Res.

[27] Murat F, Zhang R, Guizard S, Gavranovic H, Flores R, Steinbach D, Quesneville H, Tannier E, Salse J (2015). Karyotype and gene order evolution from reconstructed extinct ancestors highlight contrasts in genome plasticity of modern rosid crops. Genome Biol Evolution.

[28] DeBolt S (2010). Copy number variation shapes genome diversity in Arabidopsis over immediate family generational scales. Genome Biol Evolution.

[29] Zhou DW, Zhou J, Meng LH, Wang QB, Xie H, Guan YC, Ma ZY, Zhong Y, Chen F, Liu JQ (2009). Duplication and adaptive evolution of the COR15 genes within the highly cold-tolerant Draba lineage (Brassicaceae). Gene.

[30] Saleh B, Allario T, Dambier D, Ollitrault P, Morillon R (2008). Tetraploid citrus rootstocks are more tolerant to salt stress than diploid. Cr Biol.

[31] Fischer I, Camus-Kulandaivelu L, Allal F, Stephan W (2011). Adaptation to drought in two wild tomato species: the evolution of the Asr gene family. New Phytol.

[32] Widholm JM, Chinnala AR, Ryu JH, Song HS, Eggett T, Brotherton JE (2001). Glyphosate selection of gene amplification in suspension cultures of 3 plant species. Physiol Plantarum.

[33] Xu JH, Messing J (2009). Amplification of prolamin storage protein genes in different subfamilies of the Poaceae. Theor Appl Genet.

[34] Rizzon C, Ponger L, Gaut BS (2006). Striking similarities in the genomic distribution of tandemly arrayed genes in Arabidopsis and rice. PLoS Comput Biol.

[35] Small ID, Peeters N (2000). The PPR motif - a TPR-related motif prevalent in plant organellar proteins. Trends Biochem Sci.

[36] Lurin C, Andres C, Aubourg S, Bellaoui M, Bitton F, Bruyere C, Caboche M, Debast C, Gualberto J, Hoffmann B (2004). Genome-wide analysis of Arabidopsis pentatricopeptide repeat proteins reveals their essential role in organelle biogenesis. Plant Cell.

[37] Zsigmond L, Rigo G, Szarka A, Szekely G, Otvos K, Darula Z, Medzihradszky KF, Koncz C, Koncz Z, Szabados L (2008). Arabidopsis PPR40 connects abiotic stress responses to mitochondrial electron transport. Plant Physiol.

[38] Fujii S, Bond CS, Small ID (2011). Selection patterns on restorer-like genes reveal a conflict between nuclear and mitochondrial genomes throughout angiosperm evolution. P Natl Acad Sci USA.

[39] Foxe JP, Wright SI (2009). Signature of diversifying selection on members of the Pentatricopeptide repeat protein family in Arabidopsis lyrata. Genetics.

[40] Geddy R, Brown GG (2007). Genes encoding pentatricopeptide repeat (PPR) proteins are not conserved in location in plant genomes and may be subject to diversifying selection. BMC Genomics.

[41] McHale L, Tan XP, Koehl P, Michelmore RW (2006). Plant NBS-LRR proteins: adaptable guards. Genome Biol..

[42] Ellis J, Dodds P, Pryor T (2000). Structure, function and evolution of plant disease resistance genes. Curr Opin Plant Biol.

[43] Wu KJ, Xu T, Guo CJ, Zhang XH, Yang SH (2012). Heterogeneous evolutionary rates of Pi2/9 homologs in rice. BMC Genet..

[44] Qu SH, Liu GF, Zhou B, Bellizzi M, Zeng LR, Dai LY, Han B, Wang GL (2006). The broad-spectrum blast resistance gene Pi9 encodes a nucleotide-binding site-leucine-rich repeat protein and is a member of a multigene family in rice. Genetics.

[45] Liu G, Lu G, Zeng L, Wang GL (2002). Two broad-spectrum blast resistance genes, Pi9(t) and Pi2(t), are physically linked on rice chromosome 6. Mol Gen Genomics.

[46] Li J, Ding J, Zhang W, Zhang Y, Tang P, Chen JQ, Tian D, Yang S (2010). Unique evolutionary pattern of numbers of gramineous NBS-LRR genes. Mol Gen Genomics.

[47] McDowell JM, Simon SA (2008). Molecular diversity at the plant-pathogen interface. Dev Comp Immunol.

[48] Hanks SK, Hunter T (1995). Protein Kinases .6. The Eukaryotic Protein-Kinase Superfamily - Kinase (Catalytic) Domain-Structure And Classification. FASEB J.

[49] Manning G, Plowman GD, Hunter T, Sudarsanam S (2002). Evolution of protein kinase signaling from yeast to man. Trends Biochem Sci.

[50] Fischer I, Dievart A, Droc G, Dufayard JF, Chantret N (2016). Evolutionary dynamics of the Leucine-rich repeat receptor-like kinase (LRR-RLK) subfamily in angiosperms. Plant Physiol.

[51] Tang P, Zhang Y, Sun XQ, Tian DC, Yang SH, Ding J (2010). Disease resistance signature of the leucine-rich repeat receptor-like kinase genes in four plant species. Plant Sci.

[52] Wang GL, Song WY, Ruan DL, Sideris S, Ronald PC (1996). The cloned gene, Xa21, confers resistance to multiple Xanthomonas oryzae pv oryzae isolates in transgenic plants. Mol Plant Microbe In.

[53] Song WY, Wang GL, Chen LL, Kim HS, Pi LY, Holsten T, Gardner J, Wang B, Zhai WX, Zhu LH (1995). A receptor kinase-like protein encoded by the Rice disease resistance gene, Xa21. Science.

[54] Tan SJ, Wang D, Ding J, Tian DC, Zhang XH, Yang SH (2011). Adaptive evolution of Xa21 homologs in Gramineae. Genetica.

[55] Swanson WJ (2003). Adaptive evolution of genes and gene families. Curr Opin Genet Dev.

[56] Johnson DA, Thomas MA (2007). The monosaccharide transporter gene family in Arabidopsis and rice: a history of duplications, adaptive evolution, and functional divergence. Mol Biol Evol.

[57] Thomas JH (2006). Adaptive evolution in two large families of ubiquitin-ligase adapters in nematodes and plants. Genome Res.

[58] Hua Z, Gao Z (2019). Adaptive and degenerative evolution of the S-phase kinase-associated protein 1-like family in Arabidopsis thaliana. PeerJ.

[59] Han MV, Demuth JP, McGrath CL, Casola C, Hahn MW (2009). Adaptive evolution of young gene duplicates in mammals. Genome Res.

[60] Zhang XH, Feng Y, Cheng H, Tian DC, Yang SH, Chen JQ (2011). Relative evolutionary rates of NBS-encoding genes revealed by soybean segmental duplication. Mol Gen Genomics.

[61] Heide O, Prestrud A (2005). Low temperature, but not photoperiod, controls growth cessation and dormancy induction and release in apple and pear. Tree Physiol.

[62] Ingram J, Bartels D (1996). The molecular basis of dehydration tolerance in plants. Annu Rev Plant Phys.

[63] Thomashow MF (1999). Plant cold acclimation: freezing tolerance genes and regulatory mechanisms. Annu Rev Plant Phys.

[64] Hundertmark M, Hincha DK (2008). LEA (late embryogenesis abundant) proteins and their encoding genes in Arabidopsis thaliana. BMC Genomics..

[65] Duan JL, Cai WM (2012). OsLEA3–2, an abiotic stress induced gene of rice plays a key role in salt and drought tolerance. PLoS One.

[66] Wisniewski M, Bassett C, Norelli J, Macarisin D, Artlip T, Gasic K, Korban S (2008). Expressed sequence tag analysis of the response of apple (Malus x domestica 'Royal Gala') to low temperature and water deficit. Physiol Plantarum.

[67] Ma L, Tian T, Lin RC, Deng XW, Wang HY, Li G (2016). Arabidopsis FHY3 and FAR1 regulate light-induced myo-inositol biosynthesis and oxidative stress responses by transcriptional activation of MIPS1. Mol Plant.

[68] Ma L, Xue N, Fu XY, Zhang HS, Li G (2017). Arabidopsis thaliana far-red elongated hypocotyls3 (fhy3) and far-red-impaired response1 (far1) modulate starch synthesis in response to light and sugar. New Phytol.

[69] Mansouri M, Naghavi MR, Alizadeh H, Mohammadi-Nejad G, Mousavi SA, Salekdeh GH, Tada Y (2019). Transcriptomic analysis of Aegilops tauschii during long-term salinity stress. Funct Integr Genomic.

[70] Xu YY, Li H, Li XG, Lin J, Wang ZH, Yang QS, Chang YH (2015). Systematic selection and validation of appropriate reference genes for gene expression studies by quantitative real-time PCR in pear. Acta Physiol Plant..

[71] Vogt T, Jones P (2000). Glycosyltransferases in plant natural product synthesis: characterization of a supergene family. Trends Plant Sci.

[72] Rehman HM, Nawaz MA, Shah ZH, Ludwig-Muller J, Chung G, Ahmad MQ, Yang SH, Lee SI (2018). Comparative genomic and transcriptomic analyses of Family-1 UDP glycosyltransferase in three Brassica species and Arabidopsis indicates stress-responsive regulation. Sci Rep.

[73] Langlois-Meurinne M, Gachon CMM, Saindrenan P (2005). Pathogen-responsive expression of glycosyltransferase genes UGT73B3 and UGT73B5 is necessary for resistance to Pseudomonas syringae pv tomato in Arabidopsis. Plant Physiol.

[74] von Saint PV, Zhang W, Kanawati B, Geist B, Faus-Kessler T, Schmitt-Kopplin P, Schaffner AR (2011). The Arabidopsis Glucosyltransferase UGT76B1 conjugates Isoleucic acid and modulates plant defense and senescence. Plant Cell.

[75] Song JT, Koo YJ, Seo HS, Kim MC, Choi YD, Kim JH (2008). Overexpression of AtSGT1, an Arabidopsis salicylic acid glucosyltransferase, leads to increased susceptibility to Pseudomonas syringae. Phytochemistry.

[76] Chong J, Baltz R, Schmitt C, Beffa R, Fritig B, Saindrenan P (2002). Downregulation of a pathogen-responsive tobacco UDP-Glc:phenylpropanoid glucosyltransferase reduces scopoletin glucoside accumulation, enhances oxidative stress, and weakens virus resistance. Plant Cell.

[77] Ahrazem O, Rubio-Moraga A, Trapero-Mozos A, Climent MFL, Gómez-Cadenas A, Gómez-Gómez L (2015). Ectopic expression of a stress-inducible glycosyltransferase from saffron enhances salt and oxidative stress tolerance in Arabidopsis while alters anchor root formation. Plant Sci.

[78] Jung S, Lee T, Cheng CH, Buble K, Zheng P, Yu J, Humann J, Ficklin SP, Gasic K, Scott K (2019). 15 years of GDR: new data and functionality in the genome database for Rosaceae. Nucleic Acids Res.

[79] Edger PP, VanBuren R, Colle M, Poorten TJ, Wai CM, Niederhuth CE, Alger EI, Ou S, Acharya CB, Wang J (2018). Single-molecule sequencing and optical mapping yields an improved genome of woodland strawberry (Fragaria vesca) with chromosome-scale contiguity. GigaScience.

[80] Verde I, Jenkins J, Dondini L, Micali S, Pagliarani G, Vendramin E, Paris R, Aramini V, Gazza L, Rossini L (2017). The Peach v2. 0 release: high-resolution linkage mapping and deep resequencing improve chromosome-scale assembly and contiguity. Bmc Genomics.

[81] Zhong Y, Jia YX, Gao Y, Tian DC, Yang SH, Zhang XH (2013). Functional requirements driving the gene duplication in 12 Drosophila species. BMC Genomics..

[82] Emms DM, Kelly S (2019). OrthoFinder: phylogenetic orthology inference for comparative genomics. Genome Biol.

[83] Emms DM, Kelly S (2015). OrthoFinder: solving fundamental biases in whole genome comparisons dramatically improves orthogroup inference accuracy. Genome Biol.

[84] Koonin EV (2005). Orthologs, paralogs, and evolutionary genomics. Annu Rev Genet.

[85] Kumar S, Stecher G, Tamura K (2016). MEGA7: molecular evolutionary genetics analysis version 7.0 for bigger datasets. Mol Biol Evol.

[86] Qiao X, Li QH, Yin H, Qi KJ, Li LT, Wang RZ, Zhang SL, Paterson AH (2019). Gene duplication and evolution in recurring polyploidization-diploidization cycles in plants. Genome Biol..

[87] Larkin MA, Blackshields G, Brown NP, Chenna R, McGettigan PA, McWilliam H, Valentin F, Wallace IM, Wilm A, Lopez R (2007). Clustal W and clustal X version 2.0. Bioinformatics.

[88] Chen C, Chen H, Zhang Y, Thomas HR, Frank MH, He Y, Xia R (2020). TBtools: an integrative toolkit developed for interactive analyses of big biological data. Mol Plant.

[89] Wang Y, Tang H, Debarry JD, Tan X, Li J, Wang X, Lee TH, Jin H, Marler B, Guo H (2012). MCScanX: a toolkit for detection and evolutionary analysis of gene synteny and collinearity. Nucleic Acids Res.

