# Supplementary figures and images for: Adaptive evolution driving the young duplications in six Rosaceae species
Source: BMC Genomics. 2021 Feb 9;22:112. doi: 10.1186/s12864-021-07422-7 (PMC7871599; doi:10.1186/s12864-021-07422-7)

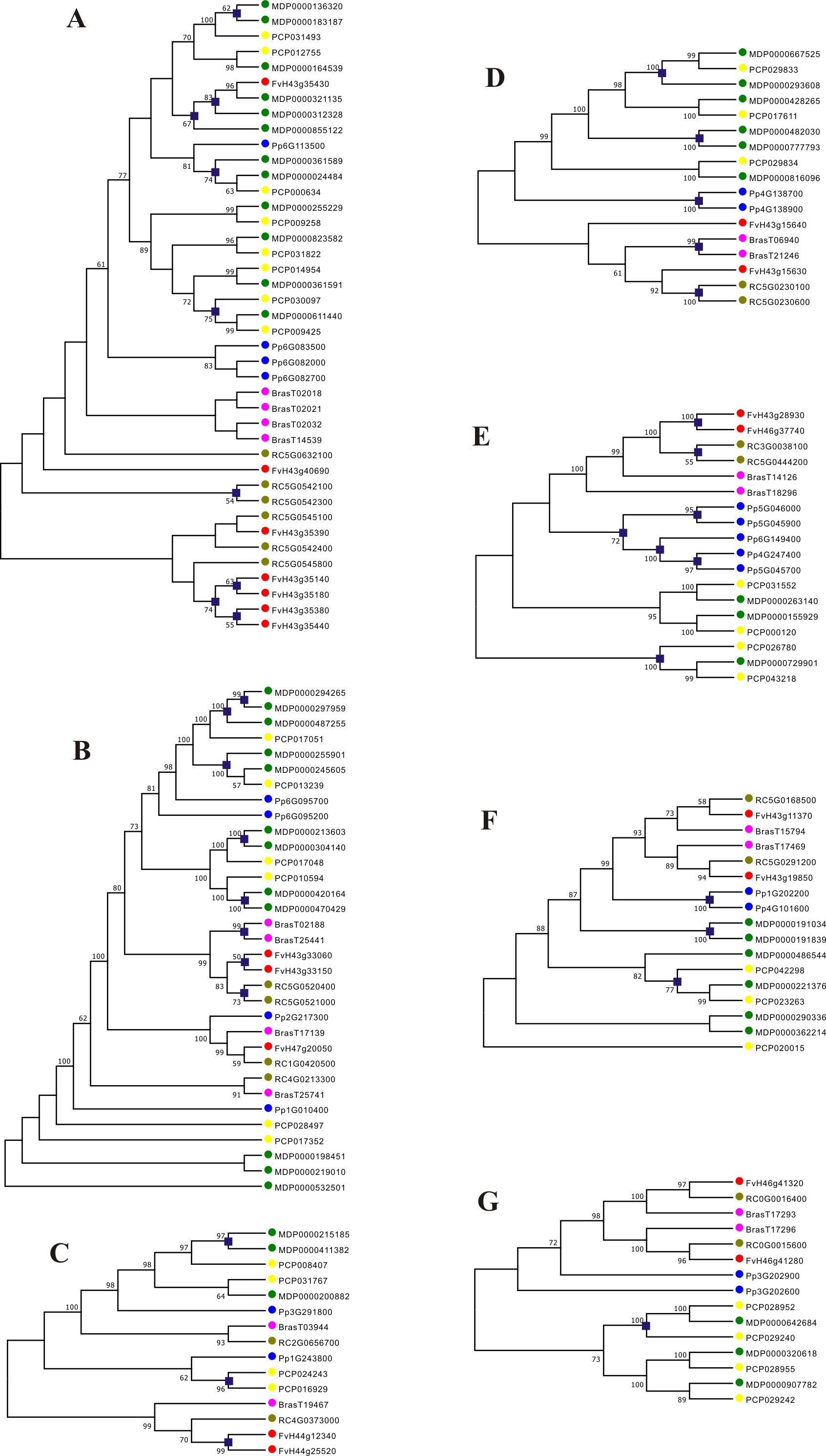

Supplement: Supplementary file 5 — Additional file 5: Figure S1. Phylogenetic trees of seven young duplicate gene families from the lineages of all six species. A: family679, B: family730, C: family1336, D: family2291, E: family4459, F: family4952, and G: family5347. Red, green, yellow, blue, brown and purple circles represent genes from F. vesca, M. x domestica, P. communis, P. persica, R. chinensis and R. occidentalis, respectively. The clade with bootstrap values larger than 50 is considered to detect the species-specific duplication and lineage-specific duplication. Blue box means species-specific duplications. [file 12864_2021_7422_MOESM5_ESM.jpg]

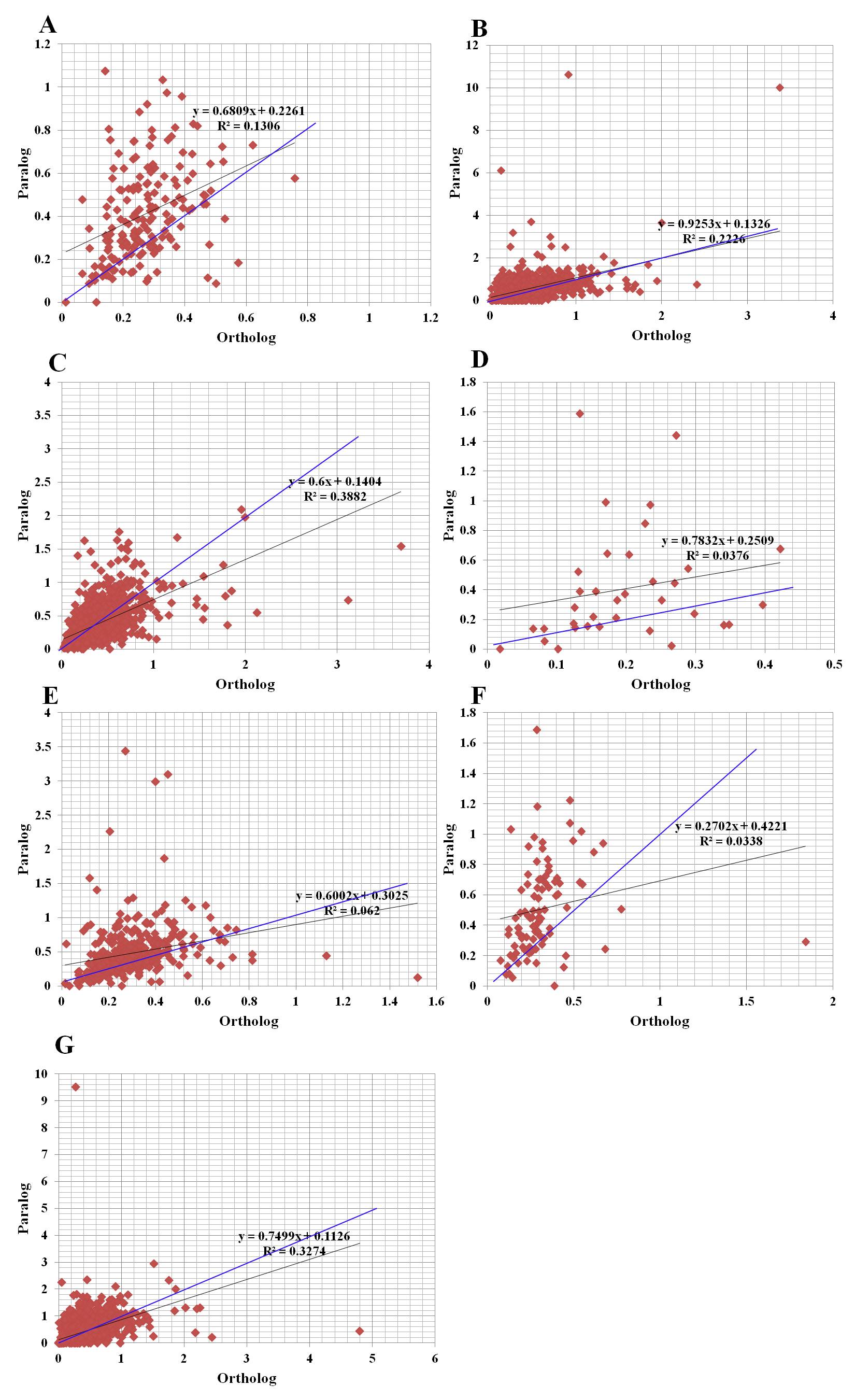

Supplement: Supplementary file 6 — Additional file 6: Figure S2. The linear analysis of Ka/Ks ratios between paralogs and orthologs in species-specific and lineage-specific expansions. A-F: the Ka/Ks values of species-specific expansions from F. vesca, M. x domestica, P. communis, P. persica, R. chinensis and R. occidentalis, respectively; G: the Ka/Ks values of lineage-specific expansions. The x-axis represents Ka/Ks values among orthologs and the y-axis means Ka/Ks values among paralogs. Black lines represent the trend line of dots and blue lines means trend lines with slope = 1. [file 12864_2021_7422_MOESM6_ESM.jpg]

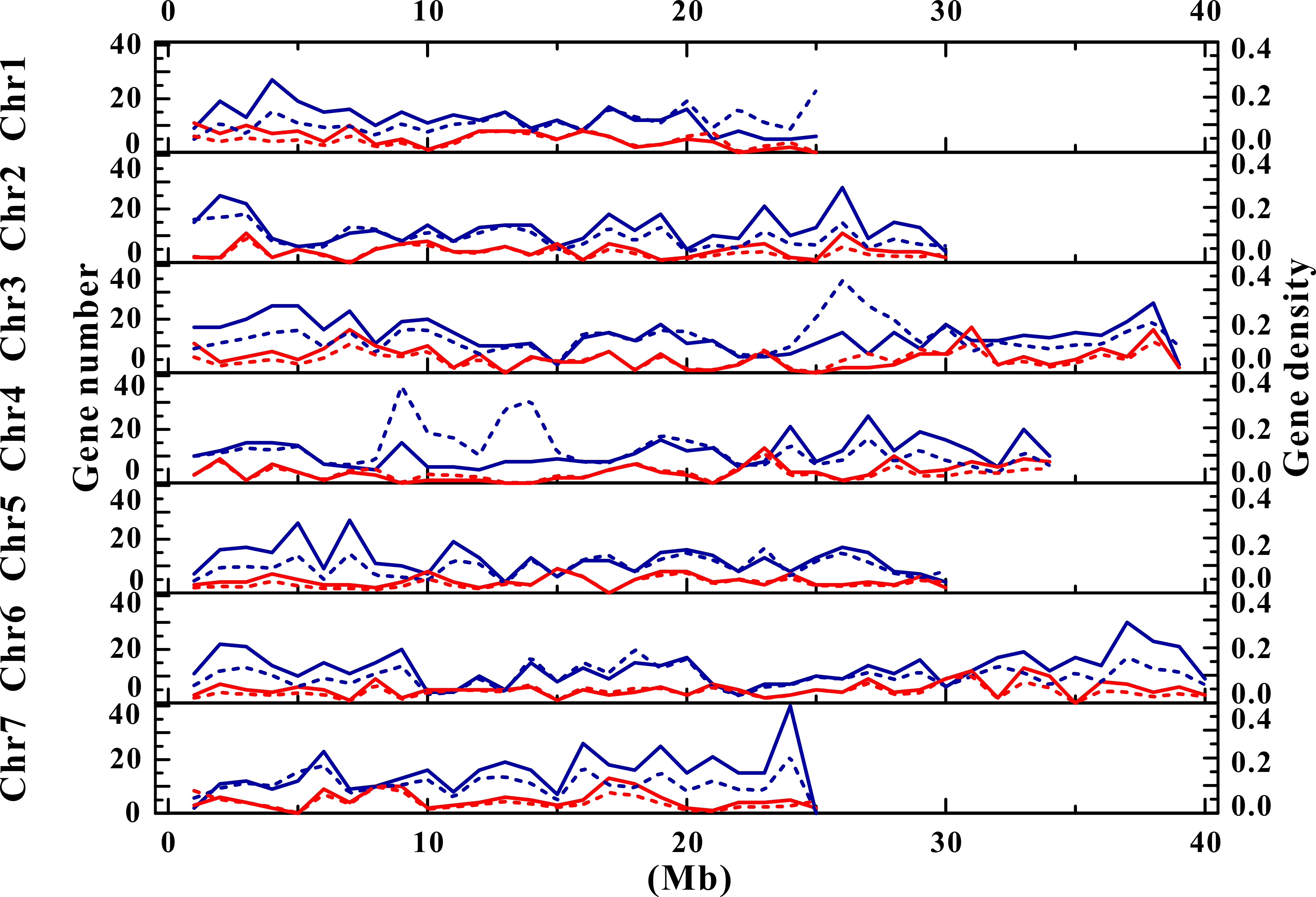

Supplement: Supplementary file 7 — Additional file 7: Figure S3. Chromosomal locations of young duplicate genes in the two types of expansions in F. vesca. Blue lines mean young duplicate genes from species-specific expansions, in which the solid ones mean gene numbers and the dotted ones are gene densities; and red lines represent young duplicate genes from lineage-specific expansions, in which the solid ones mean gene numbers and the dotted ones are gene densities. The x-axes represent the chromosomes, the left y-axes mean gene number and the right y-axes mean gene density. [file 12864_2021_7422_MOESM7_ESM.jpg]

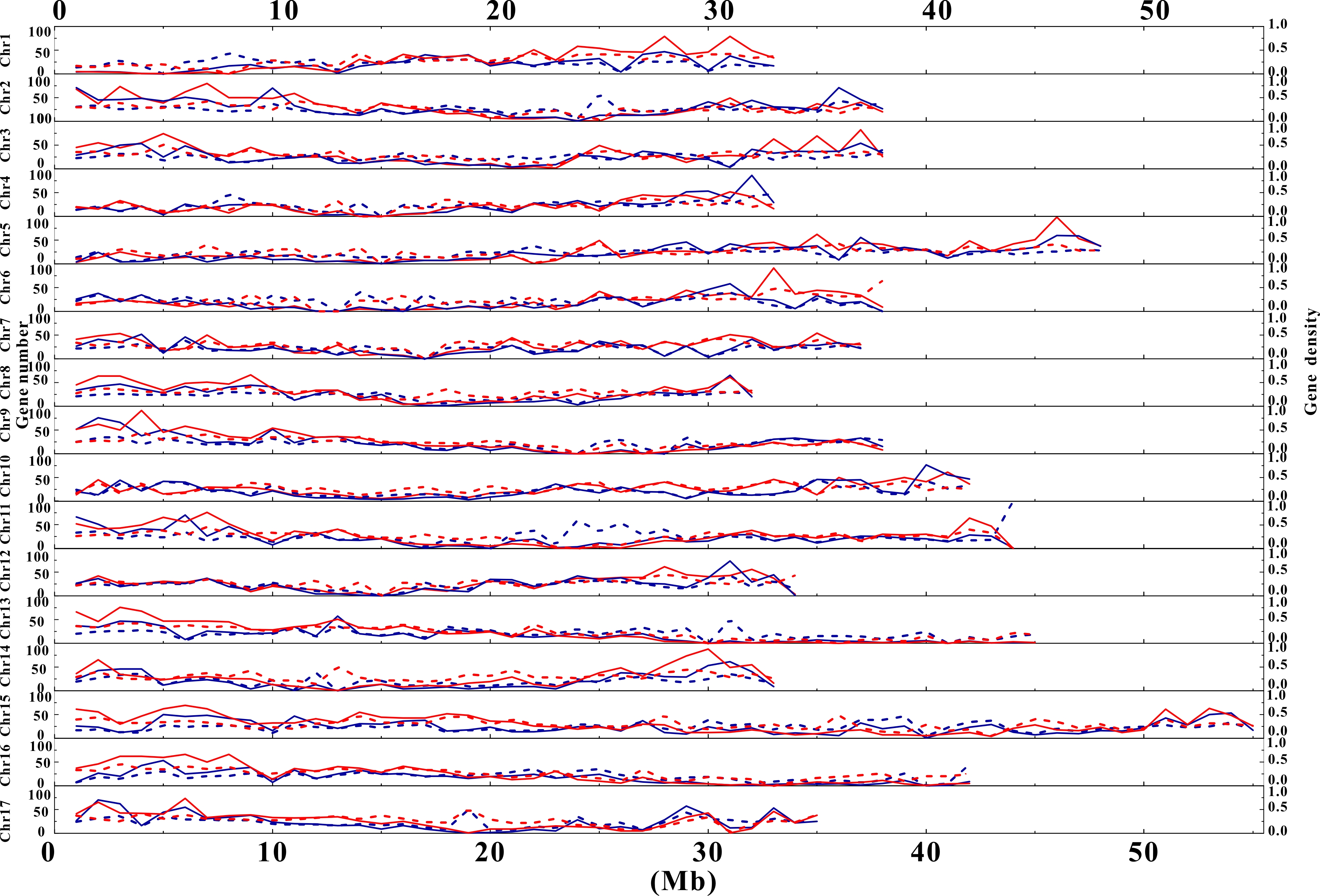

Supplement: Supplementary file 8 — Additional file 8: Figure S4. Chromosomal locations of young duplicate genes in the two types of expansions in M. x domestica. Blue lines mean young duplicate genes from species-specific expansions, in which the solid ones mean gene numbers and the dotted ones are gene densities; and red lines represent young duplicate genes from lineage-specific expansions, in which the solid ones mean gene numbers and the dotted ones are gene densities. The x-axes represent the chromosomes, the left y-axes mean gene number and the right y-axes mean gene density. [file 12864_2021_7422_MOESM8_ESM.jpg]

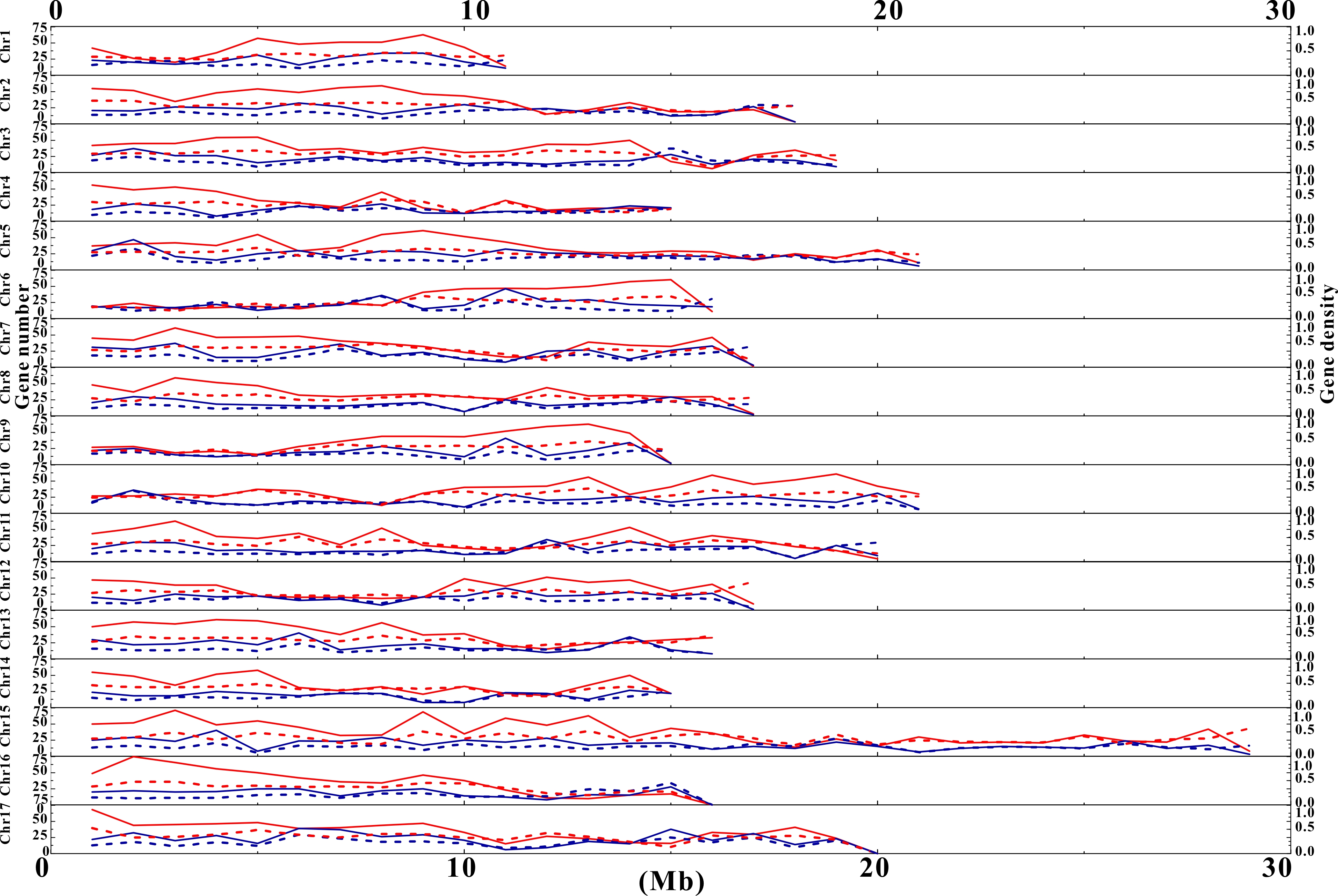

Supplement: Supplementary file 9 — Additional file 9: Figure S5. Chromosomal locations of young duplicate genes in the two types of expansions in P. communis. Blue lines mean young duplicate genes from species-specific expansions, in which the solid ones mean gene numbers and the dotted ones are gene densities; and red lines represent young duplicate genes from lineage-specific expansions, in which the solid ones mean gene numbers and the dotted ones are gene densities. The x-axes represent the chromosomes, the left y-axes mean gene number and the right y-axes mean gene density. [file 12864_2021_7422_MOESM9_ESM.jpg]

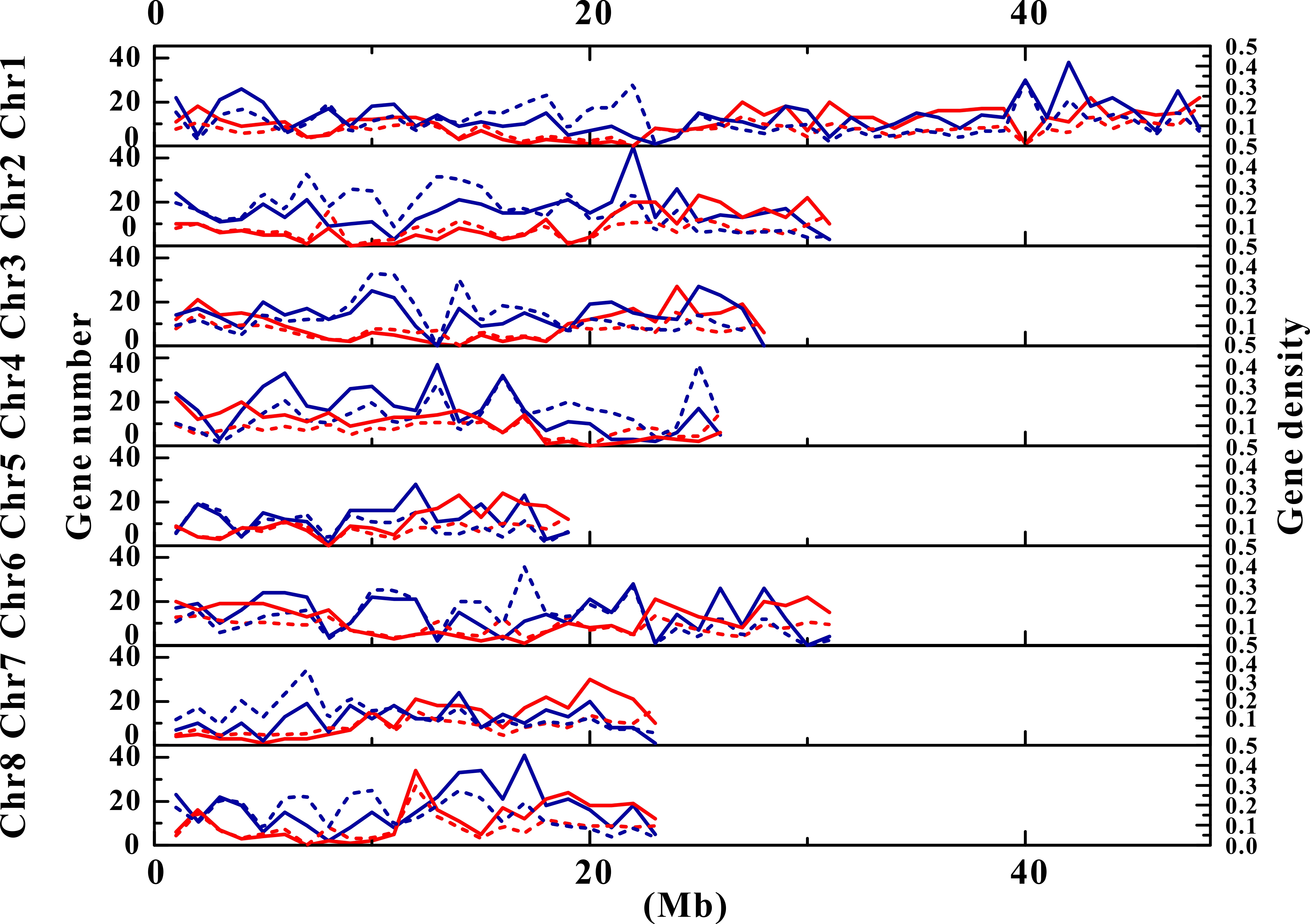

Supplement: Supplementary file 10 — Additional file 10: Figure S6. Chromosomal locations of young duplicate genes in the two types of expansions in P. persica. Blue lines mean young duplicate genes from species-specific expansions, in which the solid ones mean gene numbers and the dotted ones are gene densities; and red lines represent young duplicate genes from lineage-specific expansions, in which the solid ones mean gene numbers and the dotted ones are gene densities. The x-axes represent the chromosomes, the left y-axes mean gene number and the right y-axes mean gene density. [file 12864_2021_7422_MOESM10_ESM.jpg]

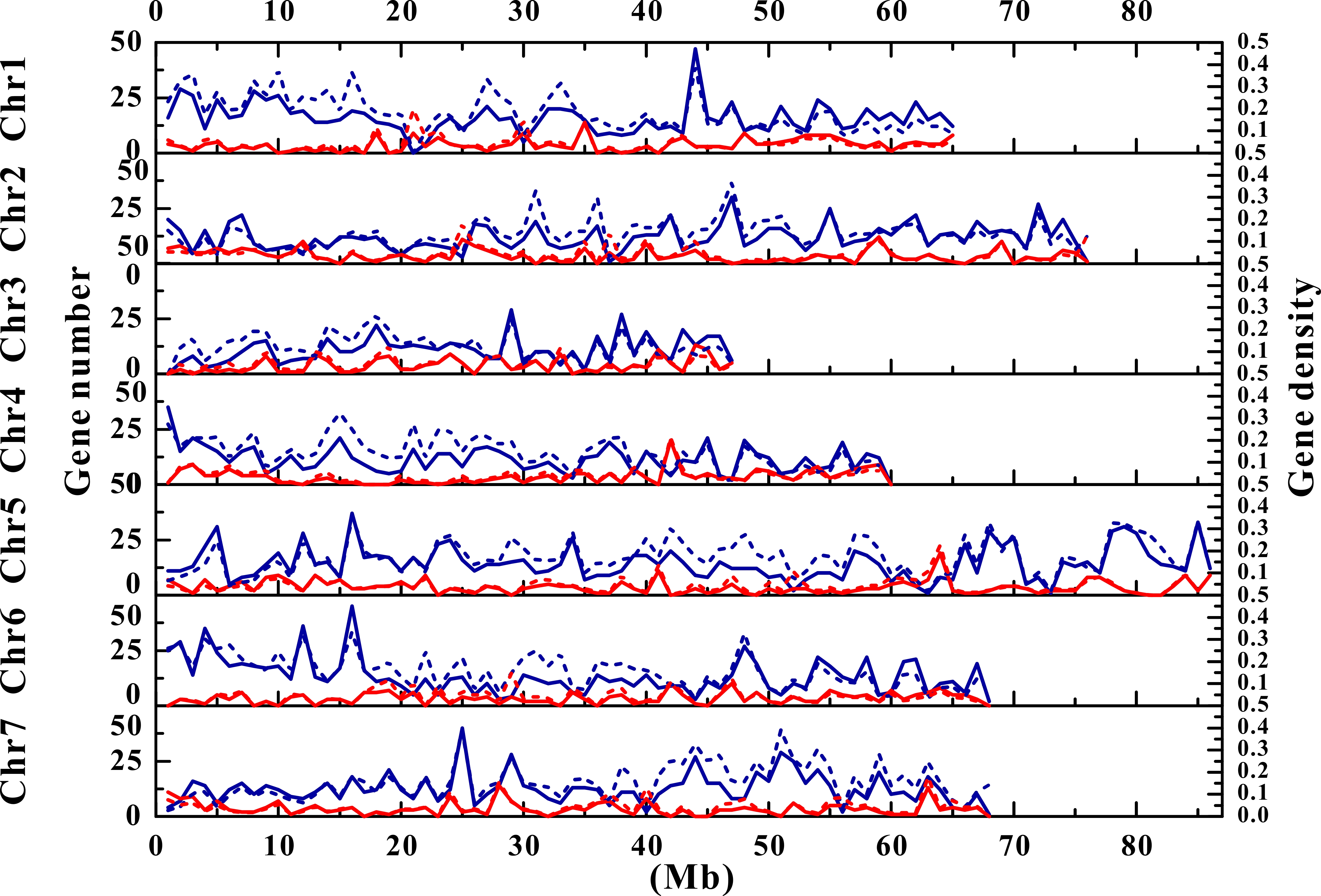

Supplement: Supplementary file 11 — Additional file 11: Figure S7. Chromosomal locations of young duplicate genes in the two types of expansions in R. chinensis. Blue lines mean young duplicate genes from species-specific expansions, in which the solid ones mean gene numbers and the dotted ones are gene densities; and red lines represent young duplicate genes from lineage-specific expansions, in which the solid ones mean gene numbers and the dotted ones are gene densities. The x-axes represent the chromosomes, the left y-axes mean gene number and the right y-axes mean gene density. [file 12864_2021_7422_MOESM11_ESM.jpg]

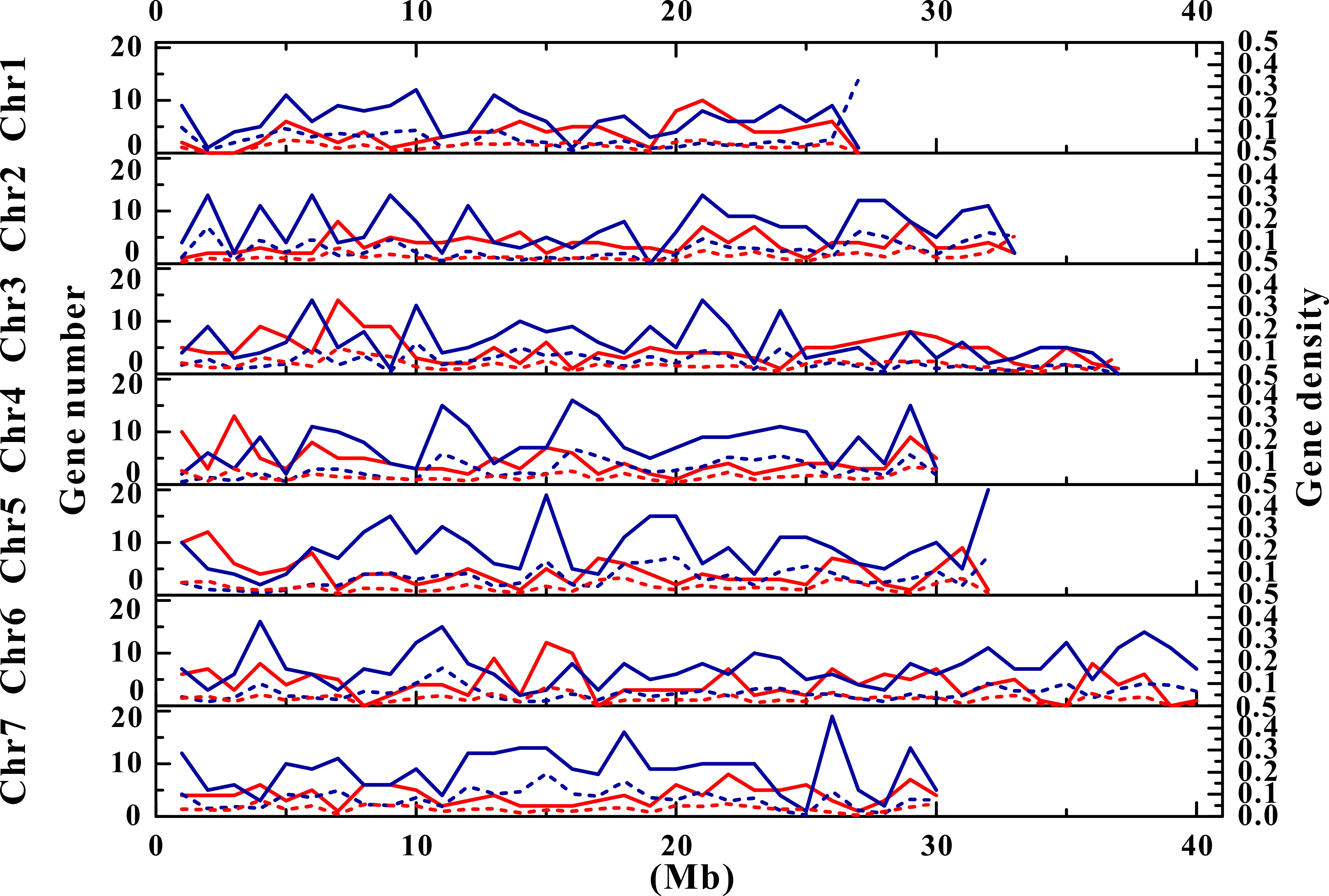

Supplement: Supplementary file 12 — Additional file 12: Figure S8. Chromosomal locations of young duplicate genes in the two types of expansions in R. occidentalis. Blue lines mean species-specific expansions, in which the solid ones mean gene numbers and the dotted ones are gene densities; and red lines represent lineage-specific expansions, in which the solid ones mean gene numbers and the dotted ones are gene densities. The x-axes represent the chromosomes, the left y-axes mean gene number and the right y-axes mean gene density. [file 12864_2021_7422_MOESM12_ESM.jpg]

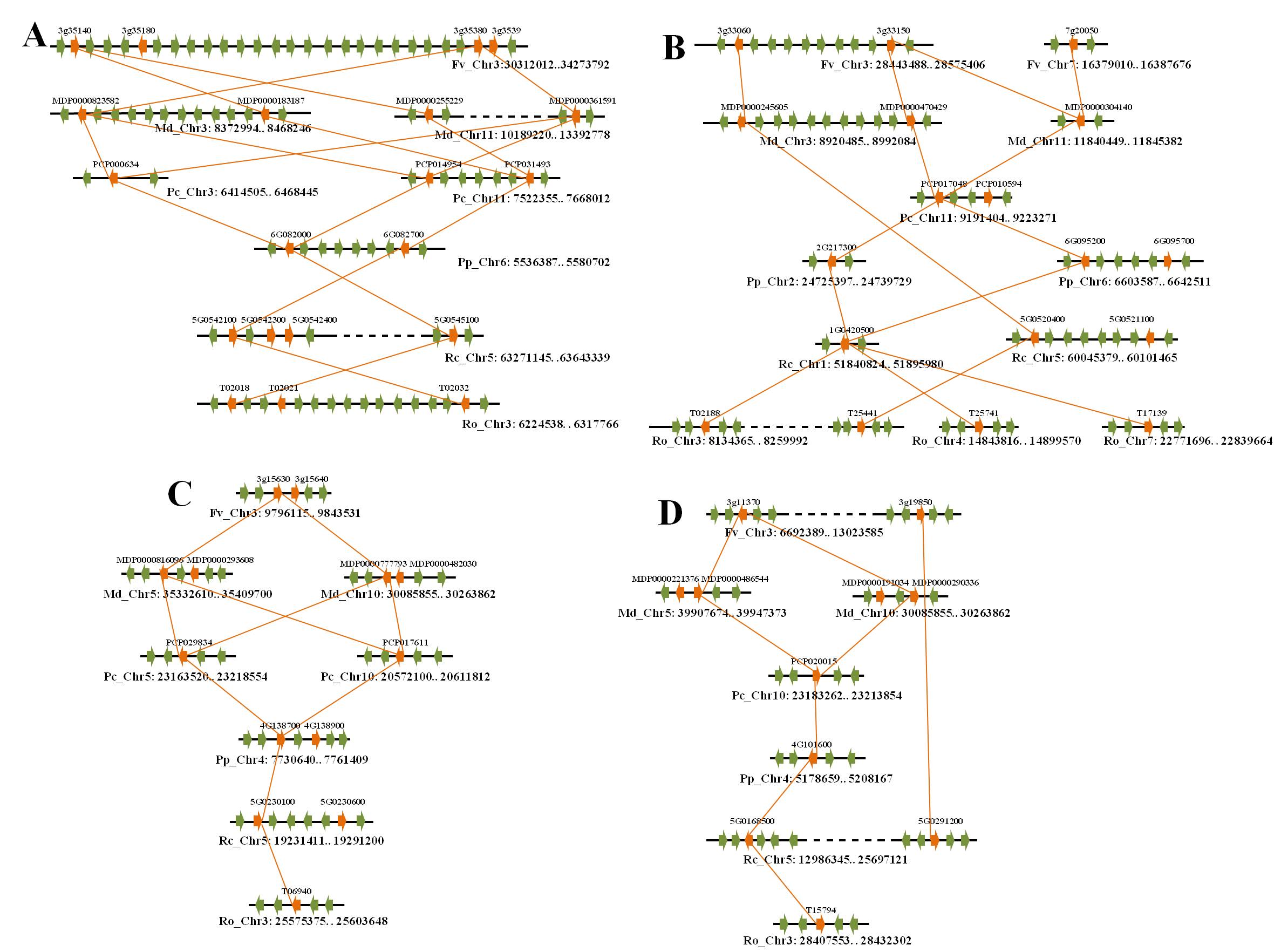

Supplement: Supplementary file 13 — Additional file 13: Figure S9. Microsynteny of young duplicate genes in four young duplicate gene families from the lineage-specific expansions co-occurring in six species. A: family679, B: family730, C: family2291 and D: family4952. Fv: F. vesca, Md: M. x domestica, Pc: P. communis, Pp: P. persica, Rc: R. chinensis and Ro: R. occidentalis. The black lines and triangles mean the chromosomal regions and the related genes on them. Orange triangles represent the gene members from the four families and the green ones are their neighbouring genes. The directions of the triangles mean the transcriptional directions of the genes. The dashed lines indicate the relative long distances on the chromosomes. The oranges lines linking the gene members represent the collinearity relationships between them. [file 12864_2021_7422_MOESM13_ESM.jpg]
